# Supplementary material for: Anti-quenching NIR-II molecular fluorophores for in vivo high-contrast imaging and pH sensing
Source: Nat Commun. 2019 Mar 5;10:1058. doi: 10.1038/s41467-019-09043-x (PMC6401027; doi:10.1038/s41467-019-09043-x)
Supplement: Supplementary file 1 — Supplementary Information [file 41467_2019_9043_MOESM1_ESM.pdf]

Supplementary Information

Anti-quenching NIR-II molecular fluorophores for *in-vivo* high-contrast imaging and pH sensing

Wang *et al*.

Supplementary Figures

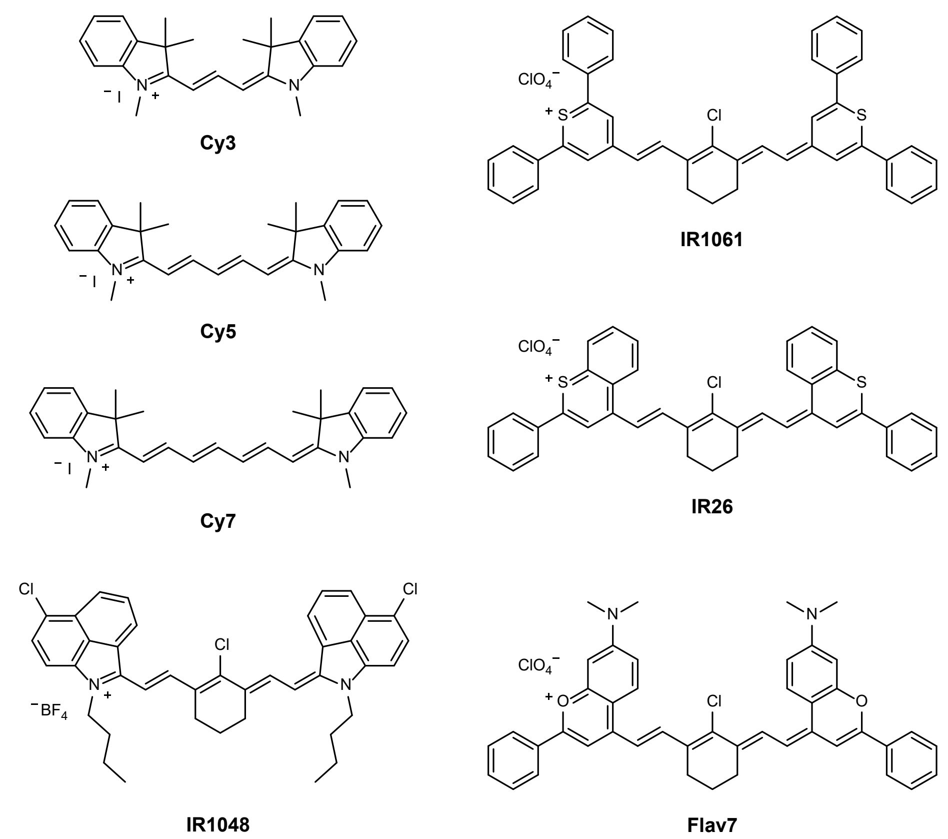

Supplementary Figure 1. Chemical structures of the compounds in Figure 1.

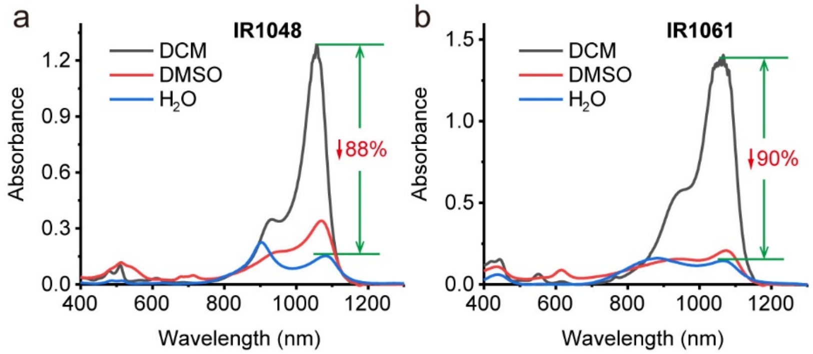

Supplementary Figure 2. Solvatochromic behavior of IR1048 and IR1061. Absorption spectra of IR1061 (**a**, 5 µM) and IR1061 (**b**, 5 µM) in different solvents. Dyes were loaded into phospholipid nanomicelles with a loading capacity of 1wt% for spectra analysis in H_2O. DCM: dichloromethane, DMSO: dimethyl sulfoxide.

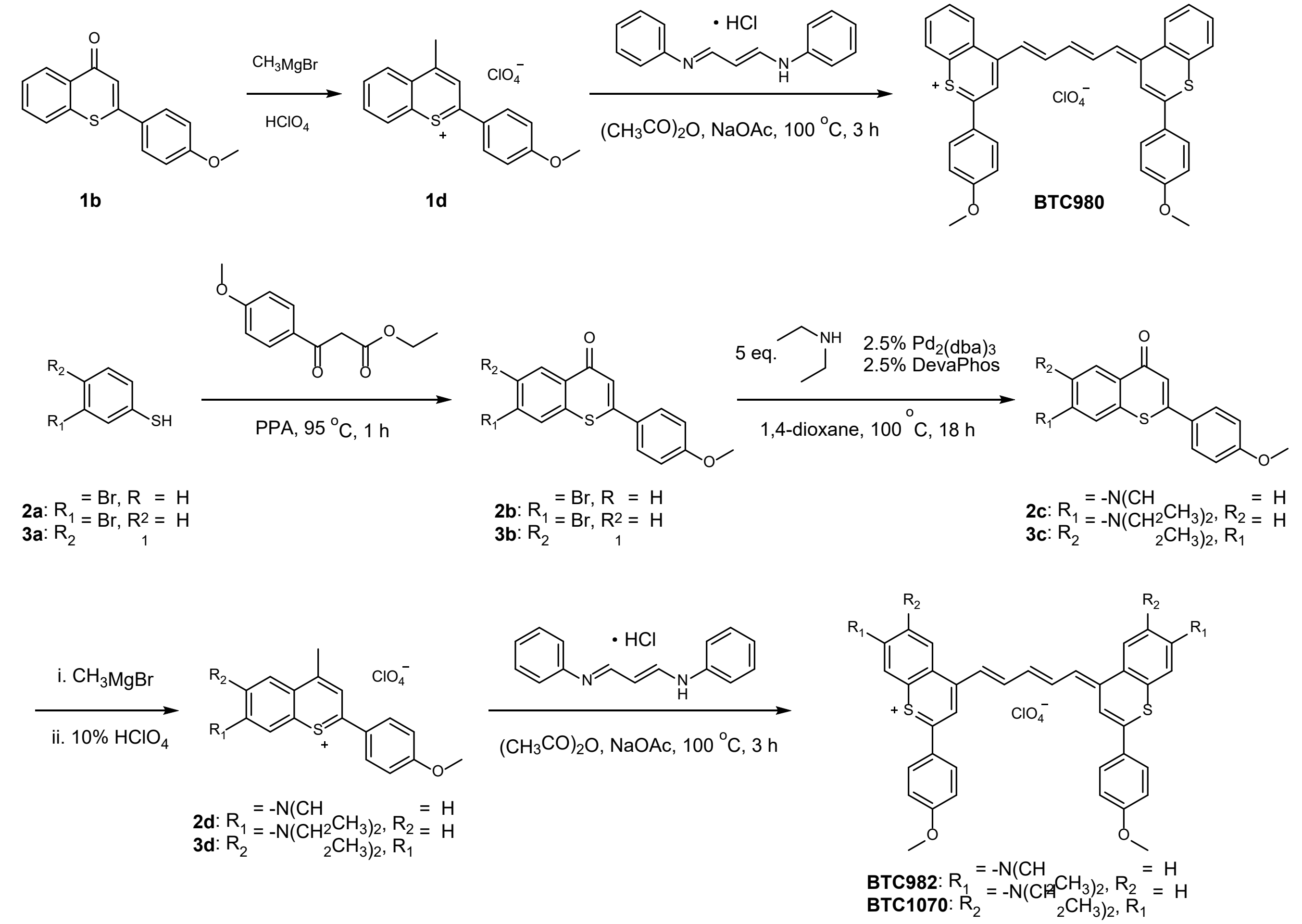

Supplementary Figure 3. Synthetic routes of BTCs.

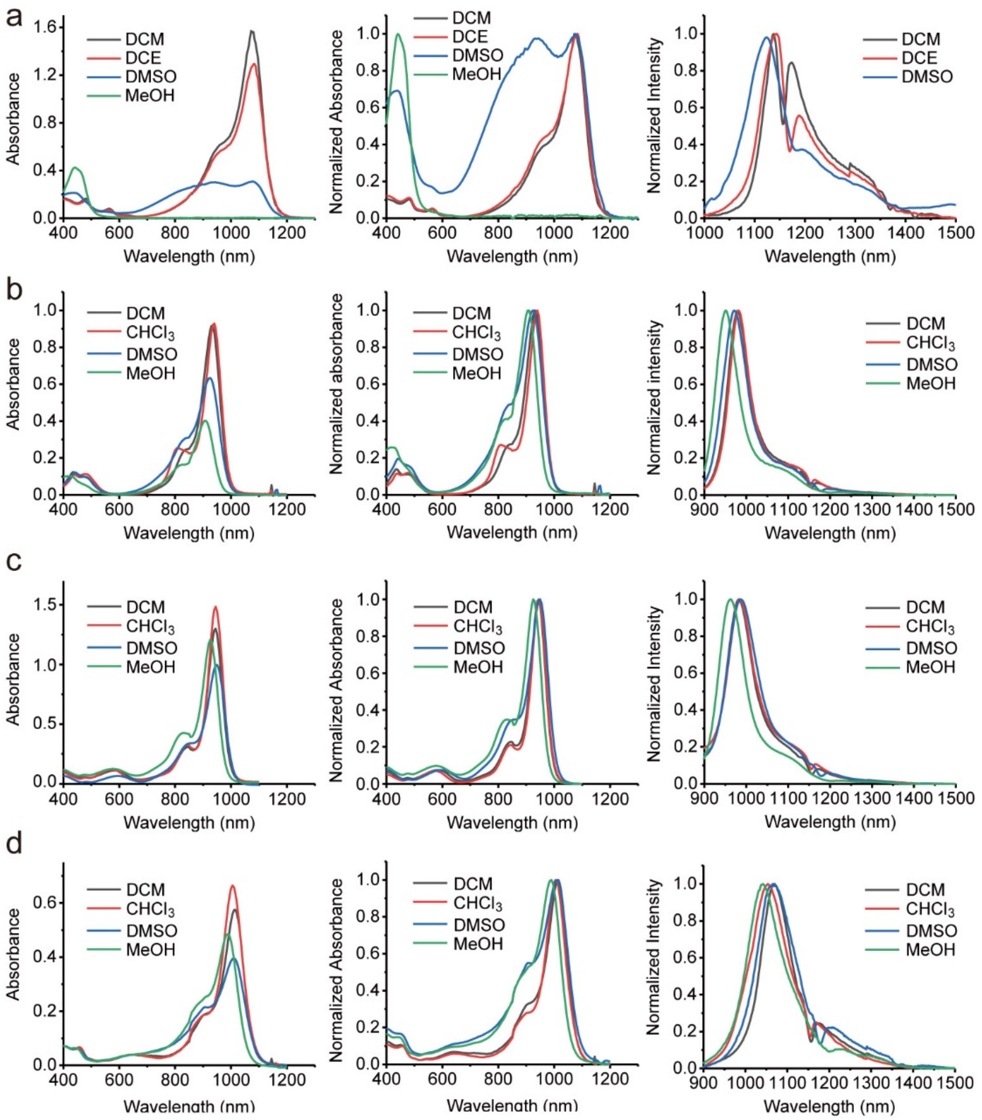

Supplementary Figure 4. Absorption and fluorescence spectra of IR26 and BTCs. a, IR-26 (10 μM). **b**, BTC980 (5 μM). **c**, BTC982 (5 μM). **d**, BTC1070 (5 μM). Excitation wavelength: 808 nm. DCM: dichloromethane, DCE: dichloro-ethane, $CHCl_3$: chloroform, DMSO: dimethyl sulfoxide, MeOH: methanol.

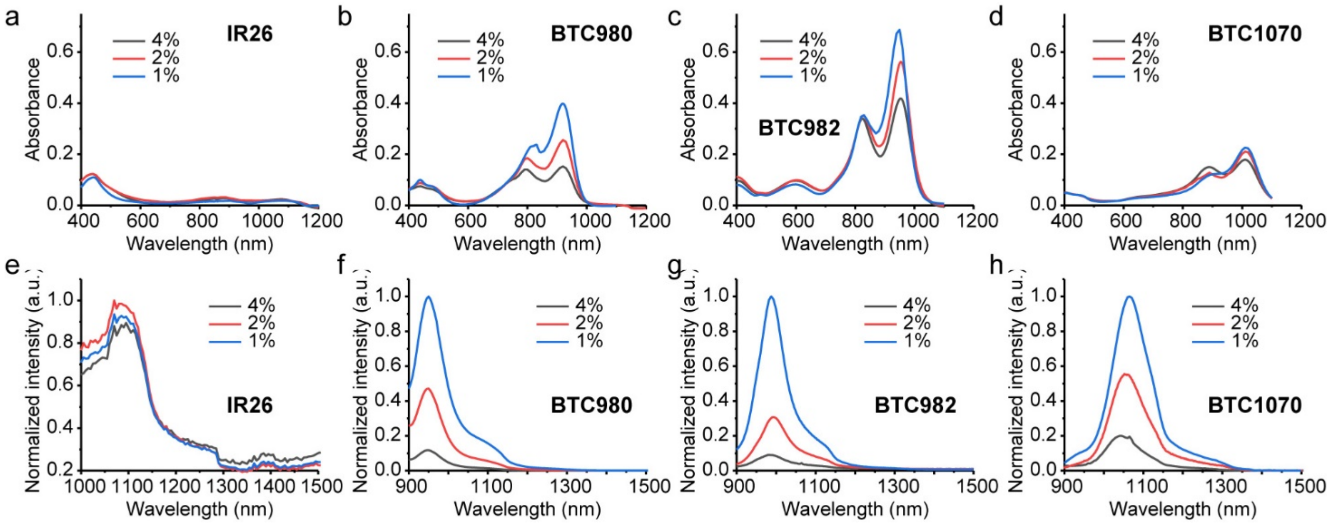

Supplementary Figure 5. Aggregation effect of IR26 and BTCs in nanomicelles. Absorption and normalized fluorescence spectra of IR26 (**a**, **e**, 5 μM), BTC980 (**b**, **f**, 5 μM), BTC982 (**c**, **g**, 5 μM) and BTC1070 (**d**, **h**, 5 μM) in PBS (pH = 7.4). Dyes were loaded into phospholipid nanomicelles with varying loading capacity of 4wt%, 2wt% and 1wt%. Excitation wavelength: 808 nm. The results show that all dyes except for IR26 exhibit loading capacity dependent absorption and fluorescence spectra, which is a direct result of dye aggregation. IR26 shows weak absorption and small changes in fluorescence at the same condition, suggesting that solvatochromism-caused quenching is the predominant factor instead of dye aggregation.

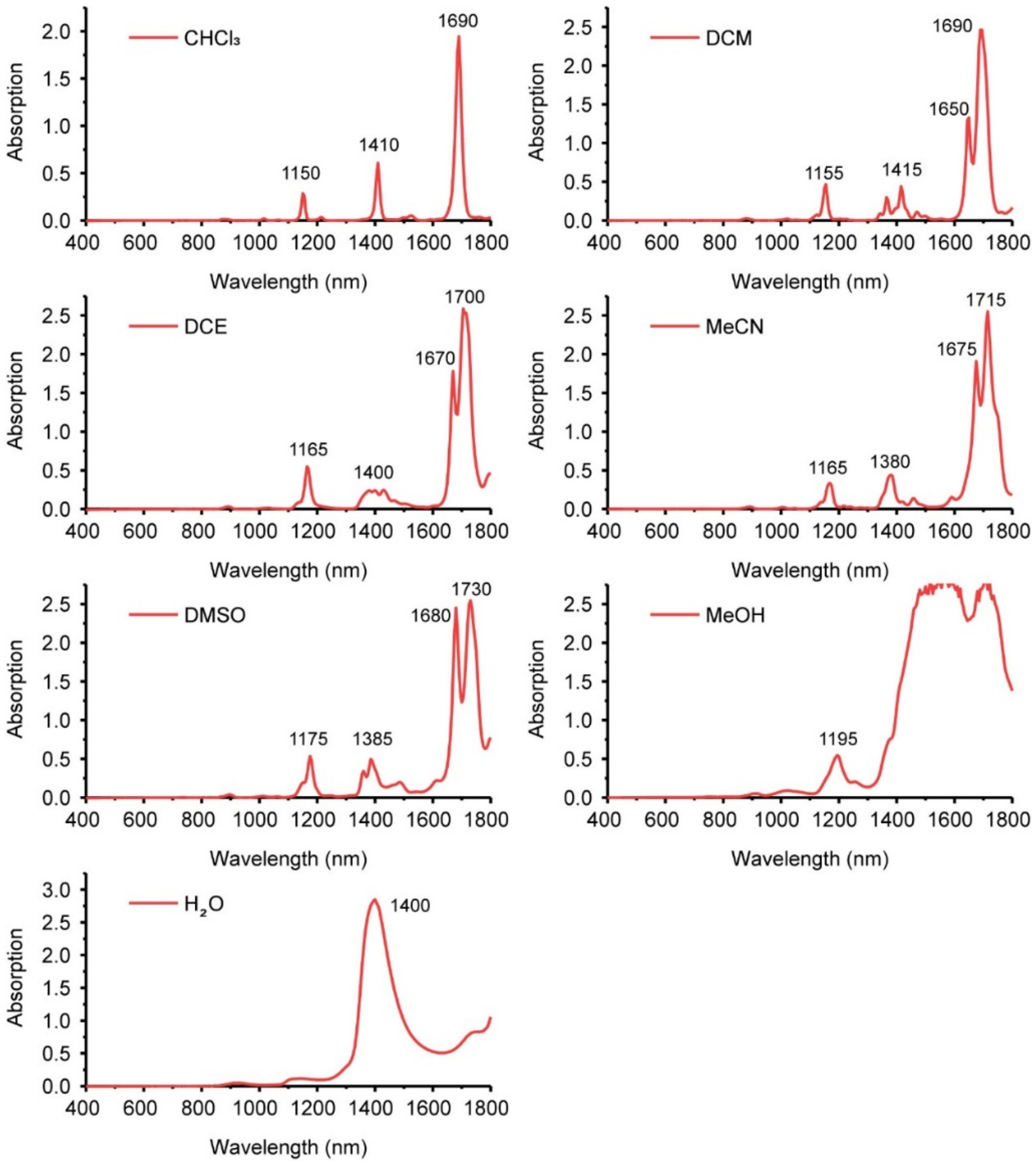

Supplementary Figure 6. Absorption spectra of various solvents. The experiments were taken with a 2 mm (1 mm for H_2O) path length cuvette. $CHCl_3$: chloroform, DCM: dichloromethane, DCE: dichloroethane, MeCN: acetonitrile, DMSO: dimethyl sulfoxide, MeOH: methanol, H_2O: deionized water. The results show that different solvents have complex fingerprint spectra in the entire NIR-II region. In our experiments, we used 10 mm path length cuvette for all the fluorescent spectra measurements. Thus, the influence of solvent absorption at 1000-1300 nm causes the different dips in fluorescent spectra (**Figure 2**). As for water, there is no sharp peaks in the absorption band at 1000-1300 nm, which explains the smooth fluorescence spectra in **Figure 5d** and **Figure 3**.

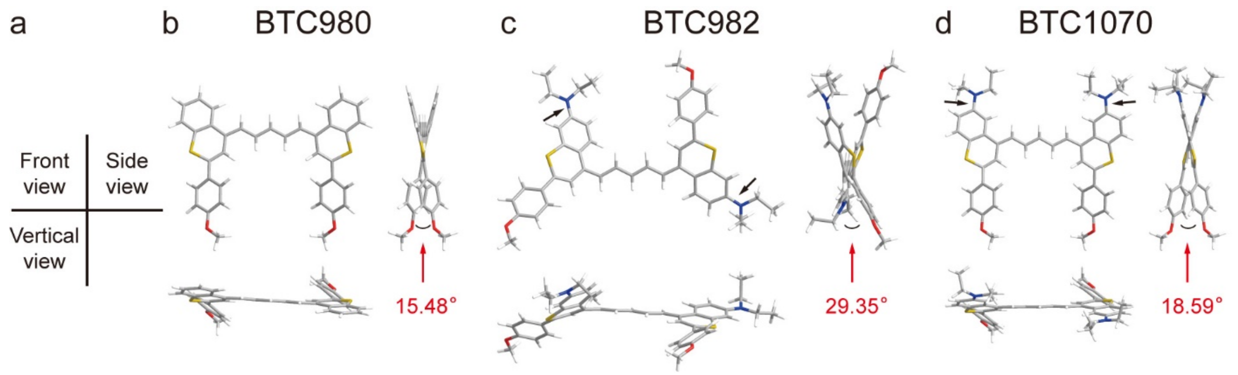

Supplementary Figure 7. Optimized molecular geometries of BTCs. Multi-view (**a**) images of optimized molecular geometries of BTC980 (**b**), BTC982 (**c**) and BTC1070 (**d**) in the ground state based on B3LYP/6-31G(d) level, showing the torsional angle between two benzothiopyrylium rings (side view). The average lengths of C-N bonds connected to the conjugated system (black arrows) are measured to be 137.3 pm for BTC982 and 138.4 pm for BTC1070.

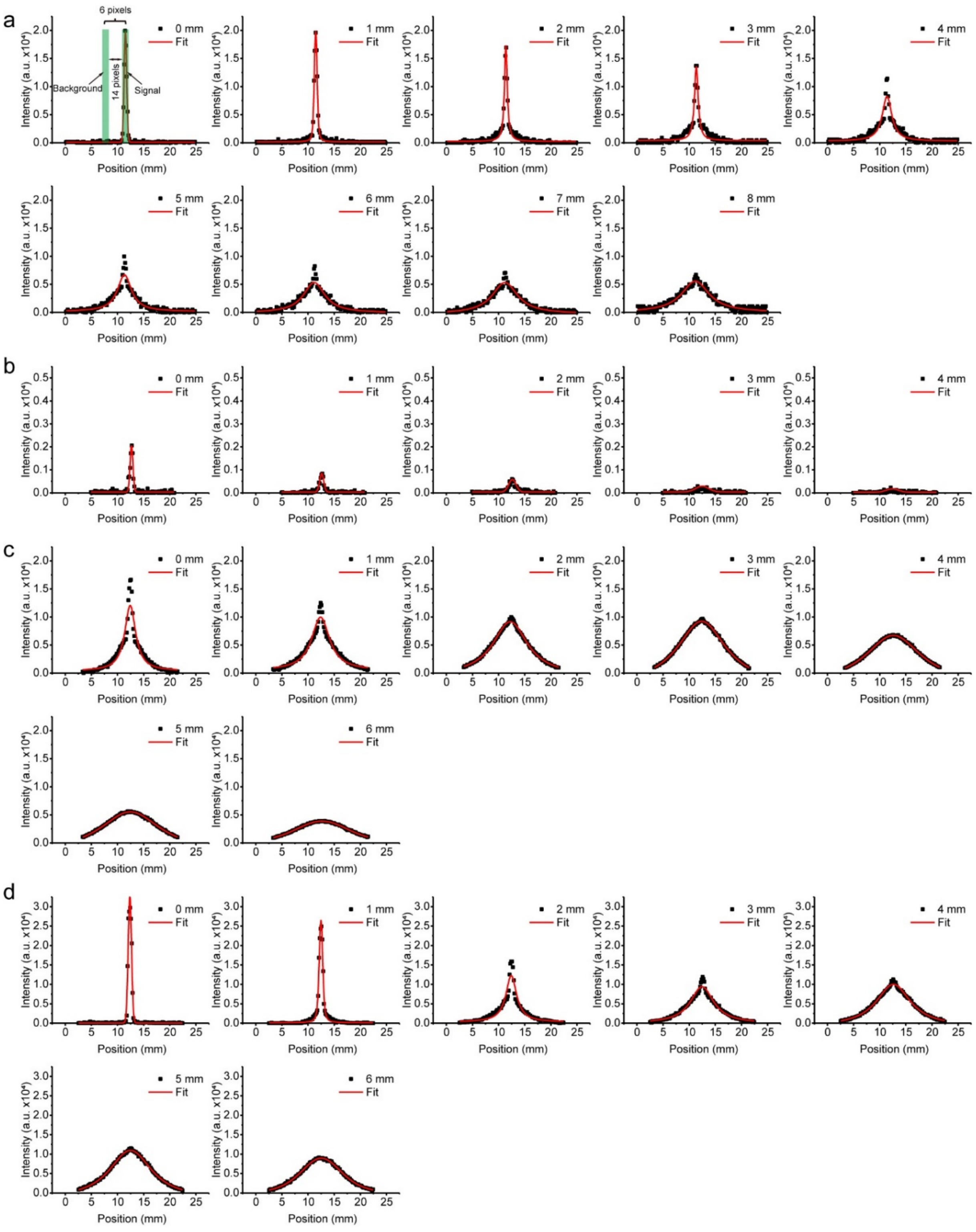

Supplementary Figure 8. Cross-sectional intensity profiles of capillary images in Figure 3b. a, BTC1070, **b**, IR26, **c**, ICG (NIR-I, 850-950 nm), **d**, ICG (NIR-II, 1000-1700 nm). FWHMs are derived from the curve fitting using Gaussian function in origin software. SBRs are calculated by dividing average signal intensity of capillary (6 pixels width) by the average background signal intensity (6 pixels width, 14 pixels apart from the capillary signal).

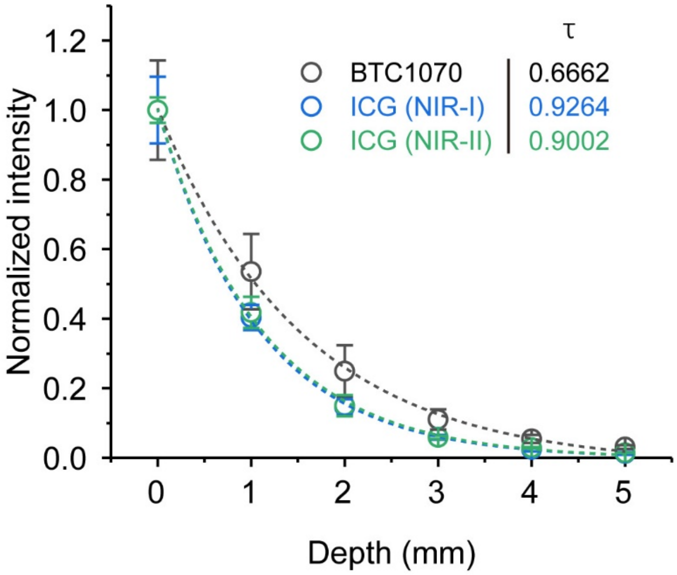

Supplementary Figure 9. Normalized capillary signals of BTC1070 and ICG in Figure 3b versus depth. Fitting the plots with single-exponential function (dash curves) gave the attenuation coefficients (τ), showing the lowest attenuation coefficient for BTC1070 imaged with 1200LP under 1064 nm excitation. The bars represent mean ± s.d. derived from n = 3 replicated measurements of every pixel of the capillaries.

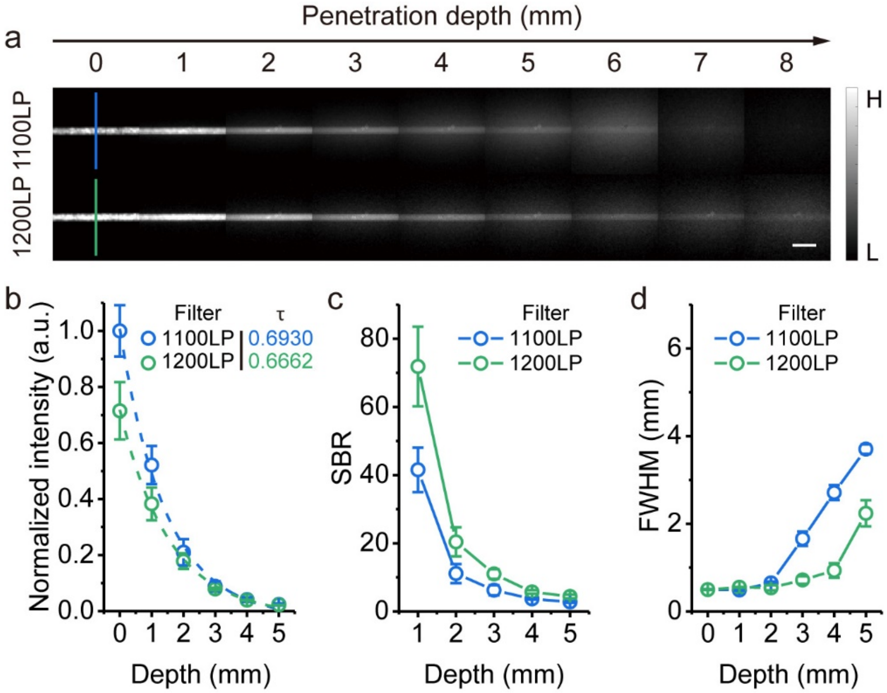

Supplementary Figure 10. Intralipid phantom imaging of BTC1070 under 1064 nm excitation. a, Fluorescence images of capillary filled with 200 μM BTC1070 in PBS (pH 7.4) immersed in 1% Intralipid with varying depth and different NIR-II filters (1100LP: 1100-1700 nm, 1200LP: 1200-1700 nm). Color bar ranges from 0 to 40000 for 1100LP channel, 0 to 20000 for 1200LP channel. The detailed imaging parameters for each image are listed in **Supplementary Table 1**. Scale bar, 2 mm. **b**, Normalized intensity of capillary as a function of depth (0-5 mm) in 1% Intralipid. Inset: the fitted attenuation coefficients (τ) at different spectra regions. **c**, Wavelength-dependent signal-to-background ratio (SBR) of capillary images as a function of depth (1-5 mm). **d**, Wavelength-dependent full-width at half-maximum (FWHM) as a function of depth (0-5 mm) in 1% Intralipid. The bars represent mean ± s.d., derived from n = 3 replicated measurements of every pixel of the capillaries for b, from n = 3 line profiles measured at different positions for c and from the uncertainty in the Gaussian fitting of feature width for d.

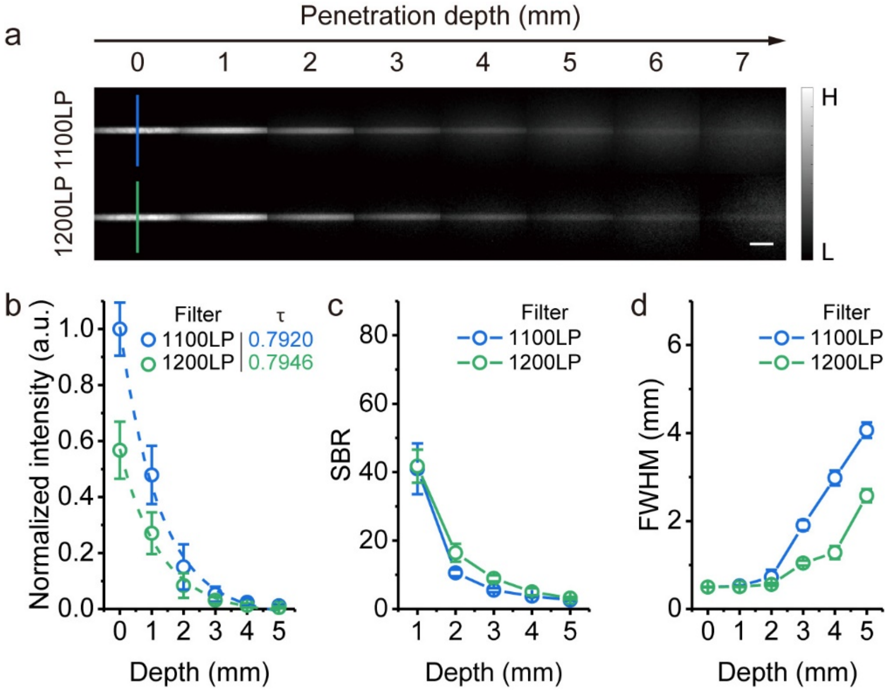

Supplementary Figure 11. Intralipid phantom imaging of BTC1070 under 915 nm excitation. a, Fluorescence images of capillary filled with 200 μM BTC1070 in PBS (pH 7.4) immersed in 1% Intralipid with varying depth and different NIR-II filters (1100LP: 1100-1700 nm, 1200LP: 1200-1700 nm). Color bar ranges from 0 to 40000 for 1100LP channel, 0 to 20000 for 1200LP channel. The detailed imaging parameters for each image are listed in **Supplementary Table 1.** Scale bar, 2 mm. **b**, Normalized intensity of capillary as a function of depth (0-5 mm) in 1% Intralipid. Inset: the fitted attenuation coefficients (τ) at different spectra regions. **c**, Wavelength-dependent signal-to-background ratio (SBR) of capillary images as a function of depth (1-5 mm). **d**, Wavelength-dependent full-width at half-maximum (FWHM) as a function of depth (0-5 mm) in 1% Intralipid. The bars represent mean ± s.d., derived from n = 3 replicated measurements of every pixel of the capillaries for b, from n = 3 line profiles measured at different positions for c and from the uncertainty in the Gaussian fitting of feature width for d.

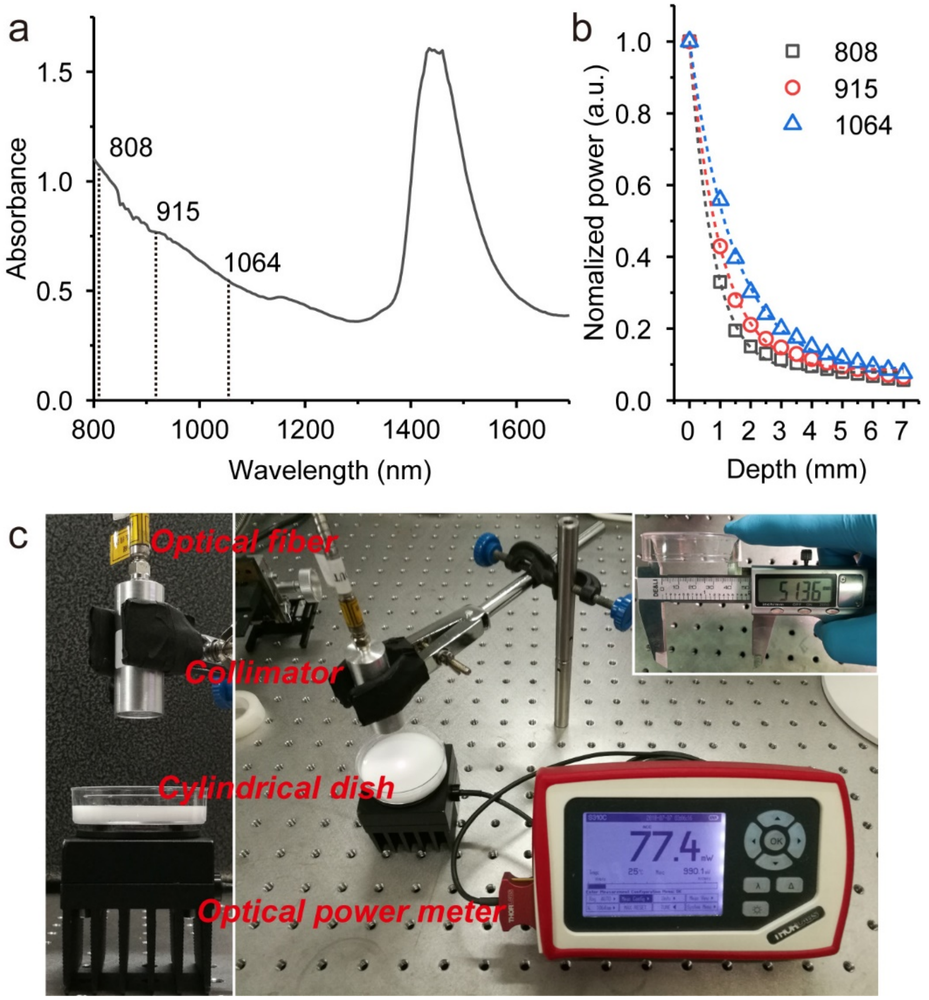

Supplementary Figure 12. Wavelength-dependent light attenuation in 1% Intralipid. **a**, Absorbance spectra of 1% Intralipid measured with a 1 mm path length cuvette, showing decreased attenuation coefficient from 808 nm to 1064 nm. **b**, Power decay of NIR laser with different wavelengths penetrating through 1% Intralipid of varying thickness, showing the attenuation trend (1064 nm< 915 nm< 808 nm) is in accord with the absorbance spectra of 1% Intralipid. **c**, Experiment setup used for evaluation the penetration depth in 1% Intralipid for several commercial excitation lasers at different wavelengths. Depth could be precisely adjusted by adding different volumes of Intralipid (1 mL of Intralipid corresponds to 0.5 mm thickness calculated from the known area of the dish).

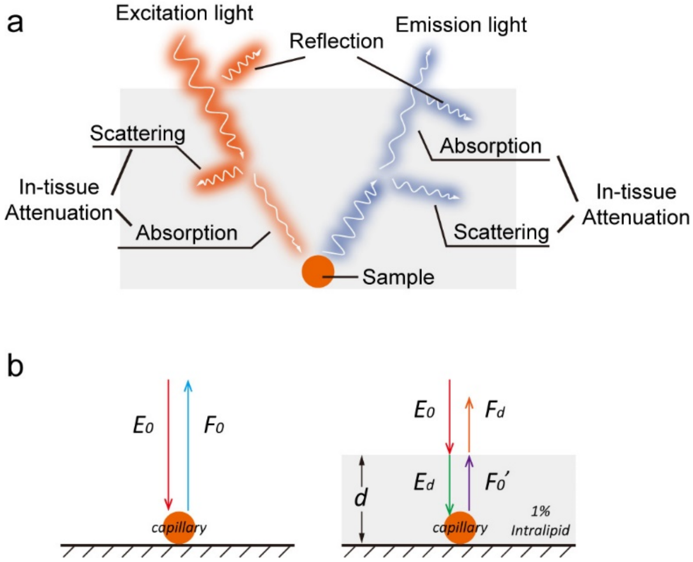

Supplementary Figure 13. The light/Intralipid interaction in phantom imaging. a, Schematic illustration shows the light/tissue interactions consist of two parts: excitation light/tissue interaction (orange) and emission light/tissue interaction (blue). Interface reflection, in-tissue scattering and absorption contribute to the loss of excitation and emission light energy, resulting in fluorescence signal attenuation in tissue. **b**, A simplified mathematical model for the description of the light/Intralipid interaction in tissue phantom imaging, showing variable parameters used in **Supplementary Equation 3-8** (**Supplementary Note 1**).

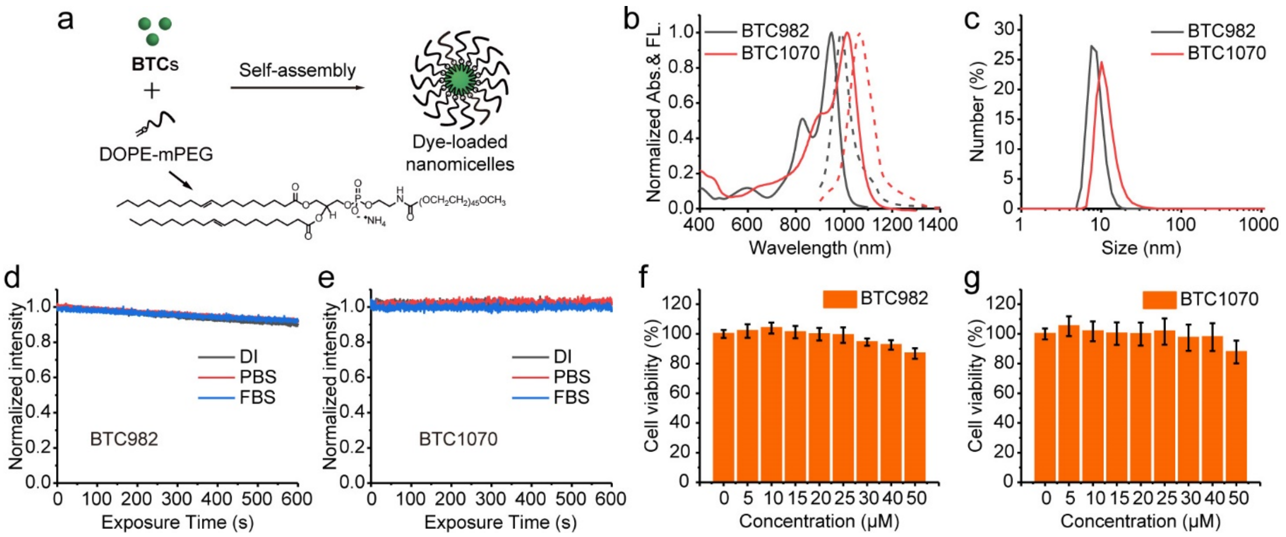

Supplementary Figure 14. Characterization of BTCs-loaded phospholipid nanomicelles. **a**, Preparation of BTCs-loaded phospholipid nanomicelles. **b**, Normalized absorption (solid) and fluorescence (dashed) spectra of BTC982- and BTC1070-loaded phospholipid nanomicelles in PBS (pH 7.4). **c**, Size distribution of BTC982- and BTC1070-loaded phospholipid nanomicelles in PBS (pH 7.4) at 37 °C determined by dynamic light scattering (DLS). **d**, **e**, Photostability of BTC982- and BTC1070-loaded phospholipid nanomicelles in a variety of biological media (DI water, 10 mM PBS and 5 % FBS) under continuous-wave laser exposure (940 nm for BTC982, 1064 nm for BTC1070) at a power density of 2.3 W cm^{-2}. **f**, **g**, Cell viability assay of BTC982- and BTC1070-loaded phospholipid nanomicelles on CaOV3 cells. The results show that the cell viability is maintained at more than 95 % for 48 h even with BTCs concentration of 40 μM, indicating the high biocompatibility of BTCs. The bars represent mean ± s.d., derived from n = 6 biologically independent measurements.

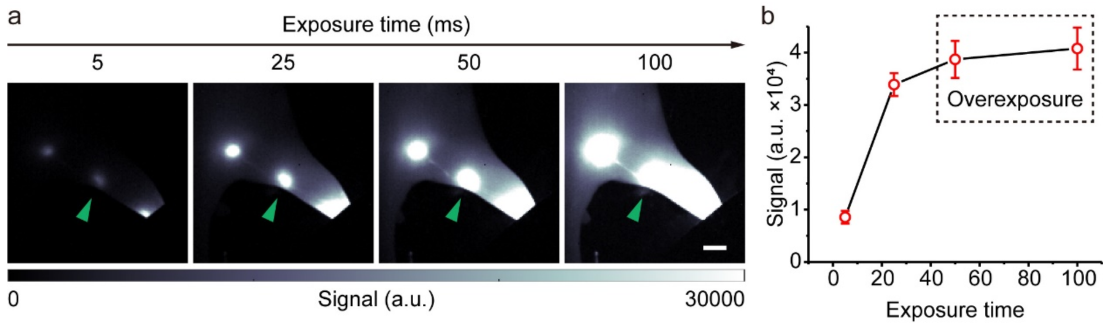

Supplementary Figure 15. In vivo lymphatic imaging with BTC982. a, Fluorescence images of lymphatic drainage in the hindlimb of nude mice at various exposure times (5, 25, 50 and 100 ms). BTC982 micelle solution (50 µL, 1.25 nmol) as contrast agent was intradermally injected into the dorsal skin of the rear paw and images were recorded at 1000-1700 nm with 1000LP filter under 915-nm (~4 mW cm^{-2}) excitation. Scale bar, 2.5 mm. **b**, Fluorescence intensity signal of popliteal lymph node (green arrows in **a**) versus exposure times. The bars represent mean ± s.d. derived from n = 3 replicated measurements of every pixel of the popliteal lymph nodes.

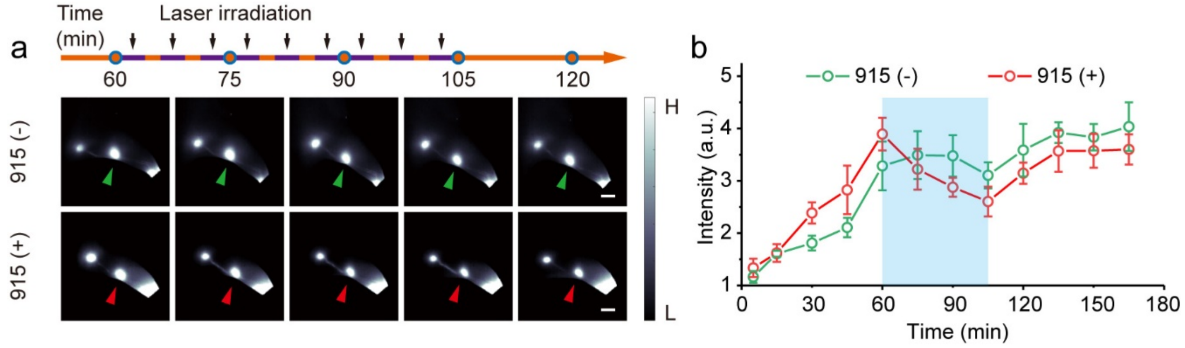

Supplementary Figure 16. In vivo photobleaching studies of BTC982. a, Fluorescence images of lymphatic drainage at different time points post-injection using BTC982. 915 (+) represents that the 915 nm laser irradiation (fluence rate is ~150 mW cm^{-2}) was conducted according to the set on the time line (purple segments on orange lines, each segment in i represents 3 min), while 915 (-) represents no laser irradiation was conducted. Scale bar, 2.5 mm. Color bar ranges from 1,000 to 30,000. The detailed imaging parameters for each image are listed in **Supplementary Table 1**. **b**, Fluorescence intensity signals of popliteal lymph nodes (green arrows/red arrows in a) versus time. Blue region: time window of laser irradiation. The bars represent mean ± s.d. derived from n = 3 biologically independent mice.

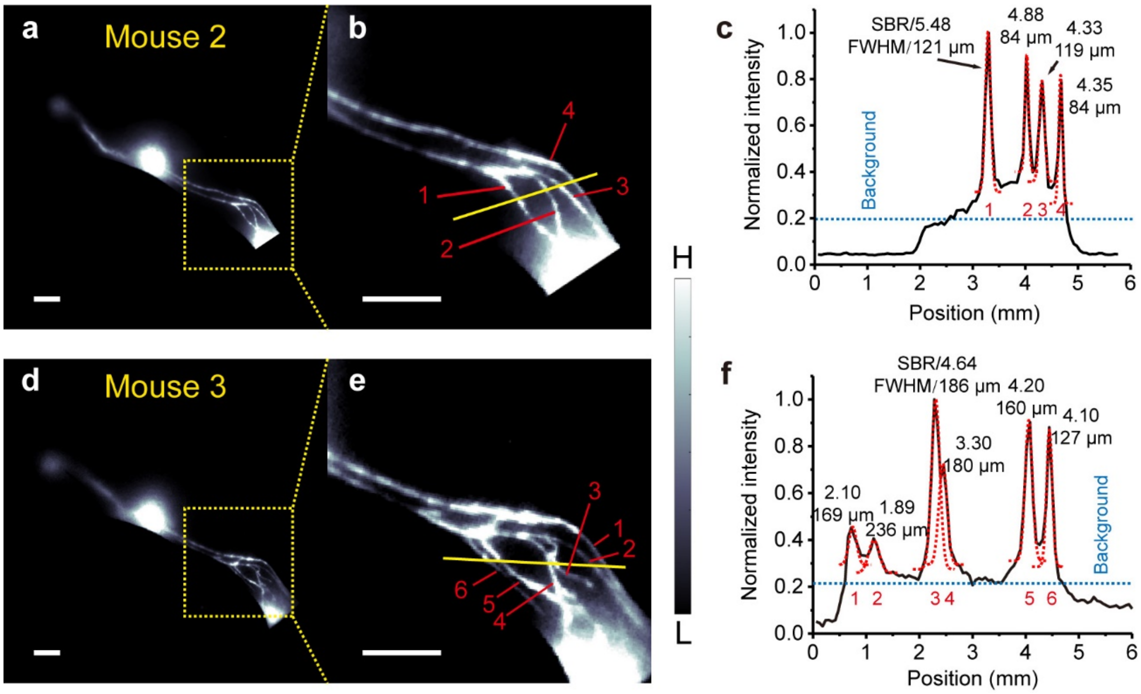

Supplementary Figure 17. In vivo lymphatic imaging with BTC1070. Fluorescence images (**a**, **d**) of lymphatic drainage on a group of nude mice (Mouse 2 and 3) using BTC1070 as contrast agent. Scale bar, 2.5 mm. Color bar ranges from 1,000 to 30,000 for a and d, 2,000 to 20,000 for b and e. Images were taken at wavelength of 1200-1700 nm under 1064 nm excitation. The detailed imaging parameters for each image are listed in **Supplementary Table 1**. **b**, **e**, high-magnification (3×) images of the ankle (yellow square in a and d), showing that 4-6 collateral lymph vessels can be resolved. Scale bar, 2.5 mm. **c**, **f**, Cross-sectional fluorescence intensity profiles (black solid) and Gaussian fit (red dotted) along the yellow bar in b and e, respectively.

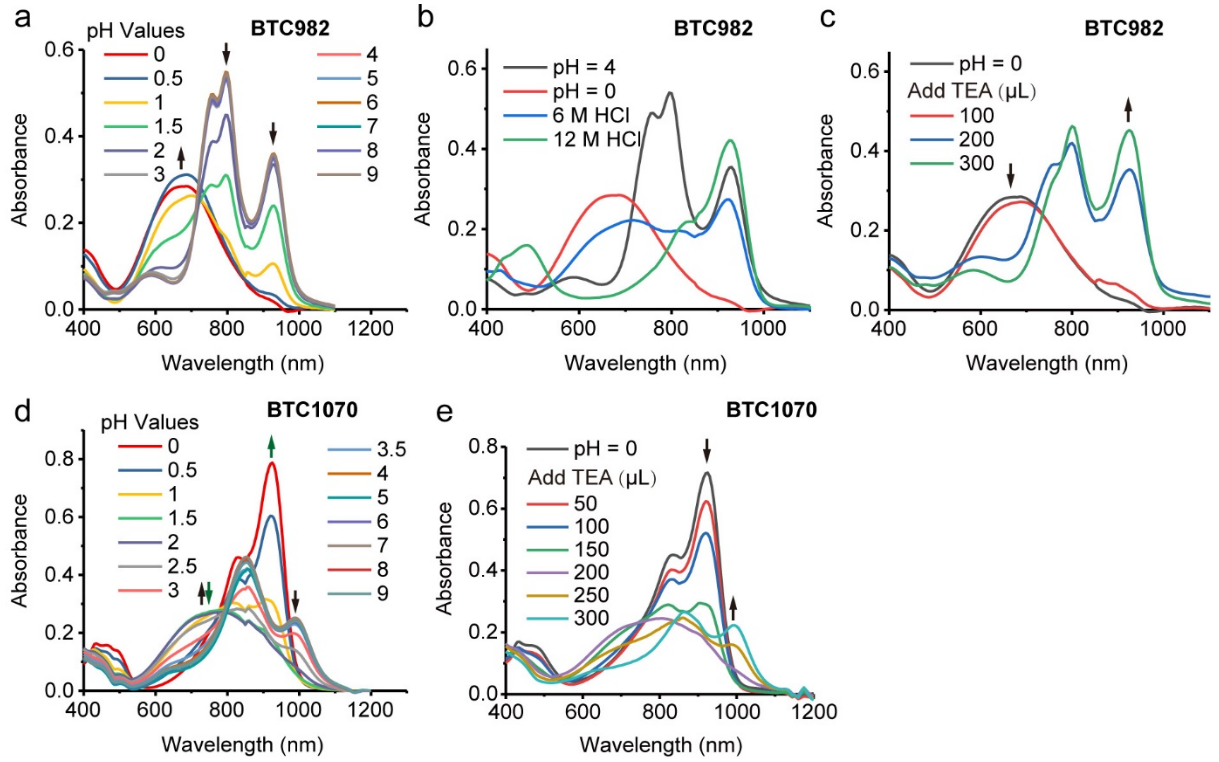

Supplementary Figure 18. pH-responsive absorption spectra of BTC982 and BTC1070 in MeCN/H_2O. a, Absorption spectra of 5 μM BTC982 in MeCN/PBS mixture (1:1, v/v) at various pH values. **b**, Absorption spectra of 5μM BTC982 in MeCN/H_2O mixture (1:1, v/v) with different acid environment. Strong acid environments were prepared by directly mixing MeCN with equal volume of 6M HCl solution or 12M HCl solution. **c**, Absorbance spectra of 5 μM BTC982 in MeCN/H_2O mixture (1:1, v/v; original pH = 0) upon addition of various amount of triethylamine (TEA). **d**, Absorption spectra of 10 μM BTC1070 in MeCN/PBS mixture (1:1, v/v) at various pH values. **e**, Absorbance spectra of 10 μM BTC1070 in MeCN/H_2O mixture (1:1, v/v; original pH = 0) upon addition of various amount of triethylamine (TEA). The results demonstrate the reversible pH responsive properties of BTC982 and BTC1070.

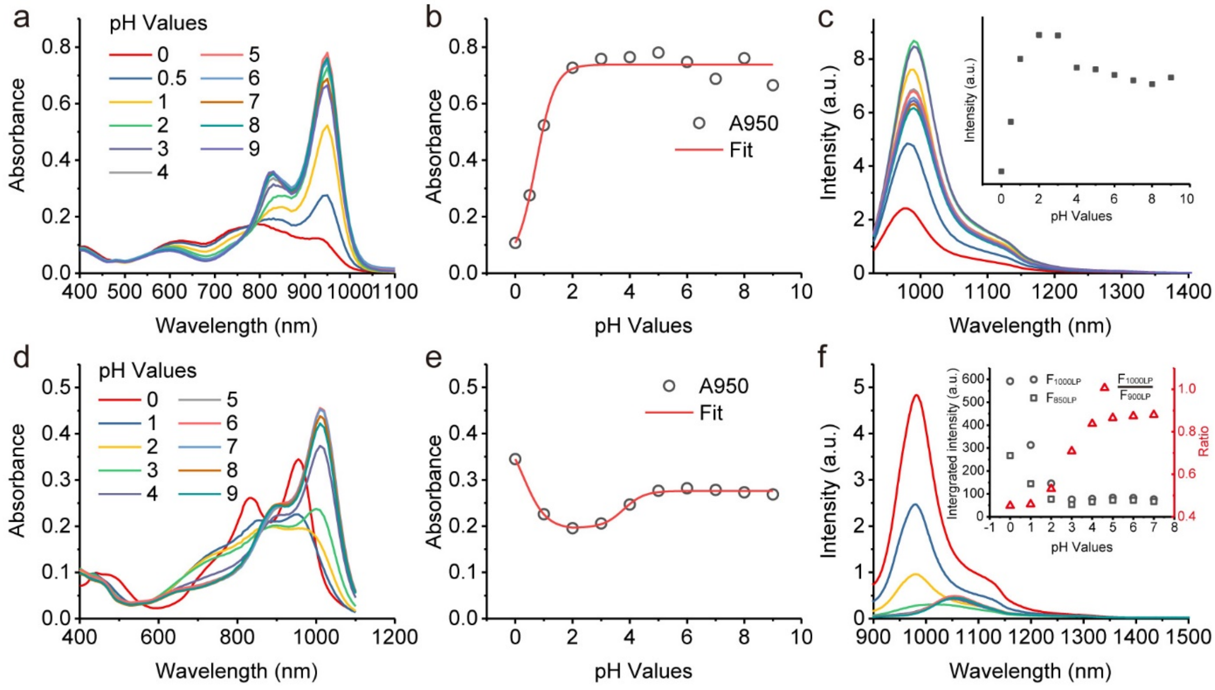

Supplementary Figure 19. pH-responsive spectra of BTC982- and BTC1070-loaded nanomicelles in H_2O. a, **d**, Absorption spectra of 5 μM BTC982- (**a**) and BTC1070- (**d**) loaded phospholipid nanomicelles in aqueous solution at various pH values. **b**, **e**, Plot of absorbance of BTC982 (**b**) and BTC1070 (**e**) at 950 nm versus pH values. Curve fitting was based on a Boltzmann function in origin software (**Supplementary Equation 2**). The p*K*a values are calculated to be 0.72 for BTC982, 0.29 and 3.81 for BTC1070. **c**, **f**, Corresponding fluorescence spectra of BTC982 (**c**) and BTC1070 (**f**). Inset in **c**: plot of integral fluorescence intensity between 940-1400 nm versus pH values. Inset in **f**: plot of integrated fluorescence intensity and ratio versus pH values.

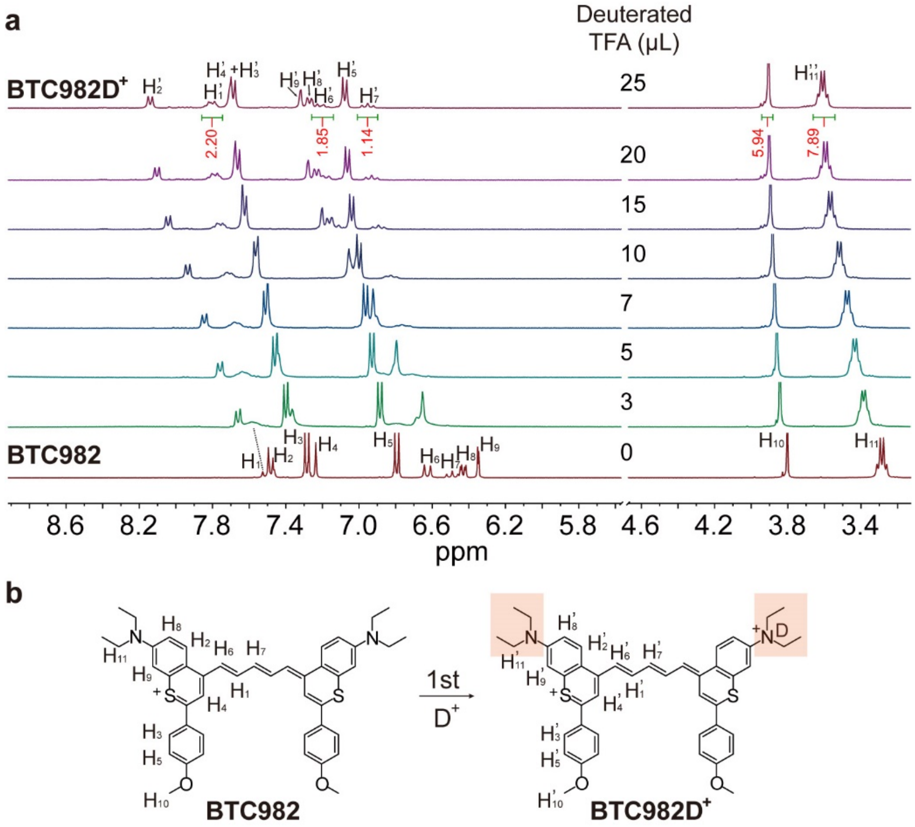

Supplementary Figure 20. Protonation mechanism for BTC982. a, ^{1}H NMR spectra (400 MHz, CD_3CN) of BTC982 upon addition of different amount of deuterated trifluoroacetic acid (D-TFA) in 0.5 mL CD_3CN. The ratio between the integrated areas of signals H_1', H_6', H_7' and H_{11}' is roughly equal to 2:2:1:8. **b**, Proposed protonation mechanism for BTC982. Orange rectangles represent protonated regions. Addition of D-TFA to BTC982 led to the downfield shifts of all proton signals belong to the conjugated system. The absorption spectra of BTC982 at the same volume ratio of TFA/MeCN (**Supplementary Figure 21a**) show only one protonation occurred during this titration. The isosbestic point at ~755 nm indicates that the first protonation of BTC982 involves an equilibrium between two species. Based on these results, the first protonation of BTC982 mainly occurs on one of the nitrogen atoms. Because the acid of TFA/MeCN mixture cannot reach the second p*K*a of BTC982, we cannot observe the second protonation, but it most probably occurs on another nitrogen atom according to the absorption spectra in 12 M HCl (**Supplementary Figure 18b**).

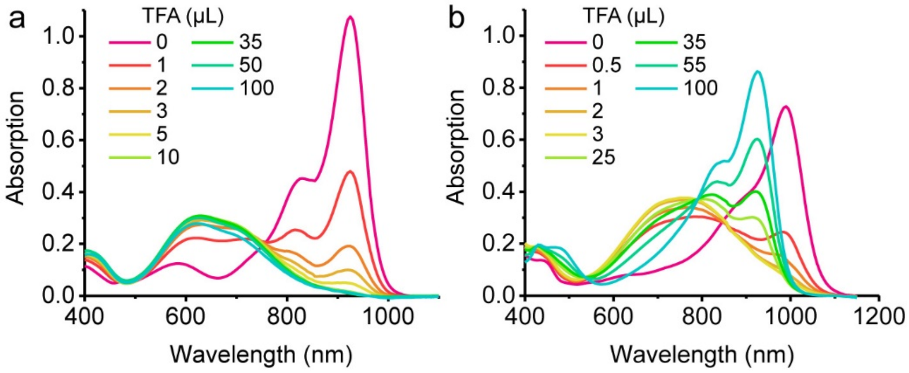

Supplementary Figure 21. Absorption spectra of BTC982 and BTC1070 in MeCN upon the addition of TFA. a, BTC982. **b**, BTC1070. The volume of MeCN is 2 mL. TFA: Trifluoroacetic acid.

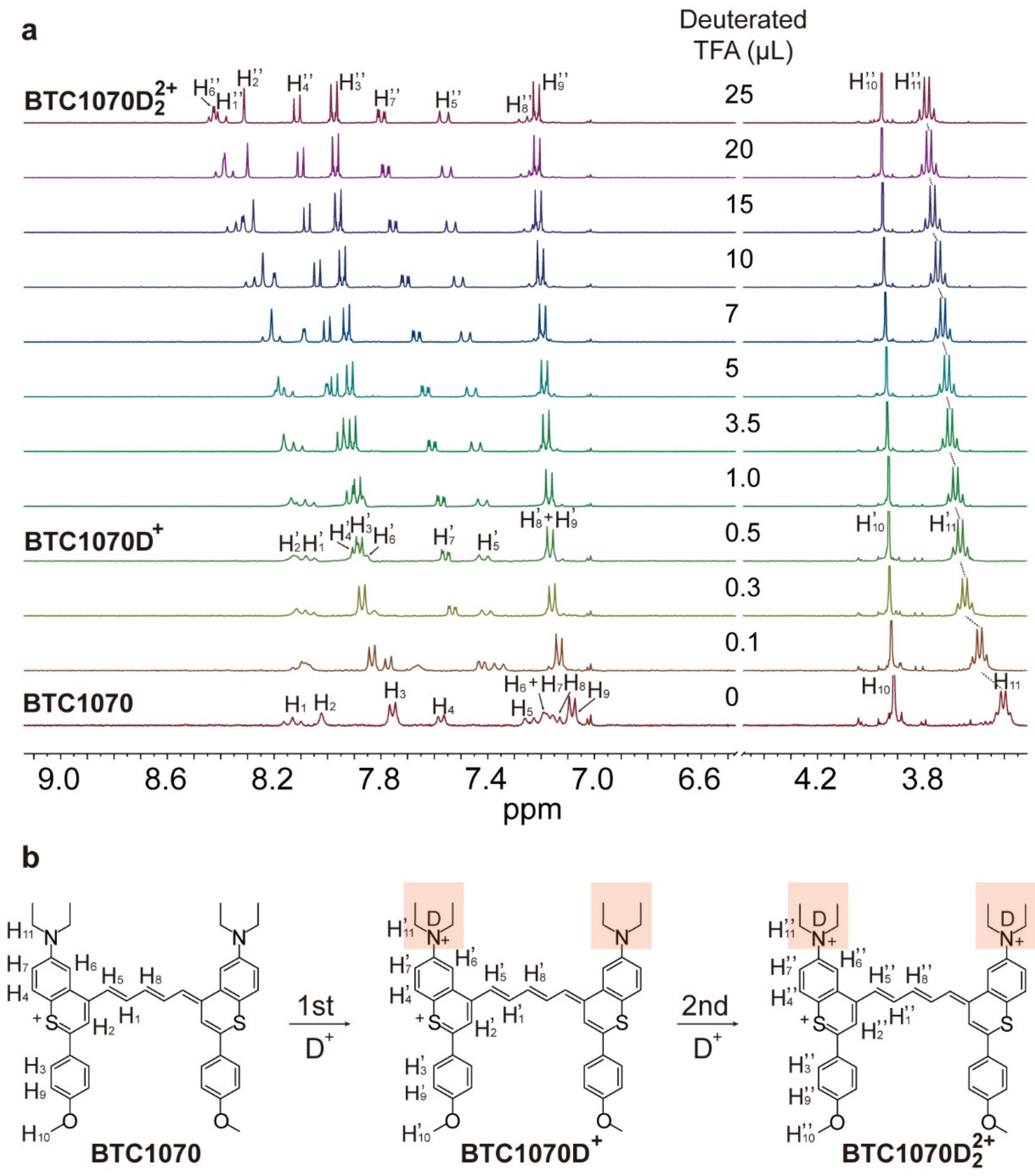

Supplementary Figure 22. Protonation mechanism for BTC1070. a, ^{1}H NMR spectra (400 MHz, CD_3CN) of BTC1070 upon addition of different amount of deuterated TFA in 0.5 mL CD_3CN. **b**, Proposed protonation mechanism for BTC1070. Orange rectangles represent protonated regions. The absorption spectra of BTC1070 in MeCN upon addition of TFA (**Supplementary Figure 21b**) show two protonation processes with two isosbestic points at ~850 nm and ~790 nm, respectively, revealing that both protonation steps involve equilibriums between two species. Additionally, in ^{1}H NMR titrations, methylene protons of diethylamino groups show downfield shifts ($\Delta\delta[H_{11}] = 0.29$ ppm), revealing the deshielding effect of protonated nitrogen atoms. Combining all the results, the protonation of BTC1070 is indeed occurred on the two nitrogen atoms step by step.

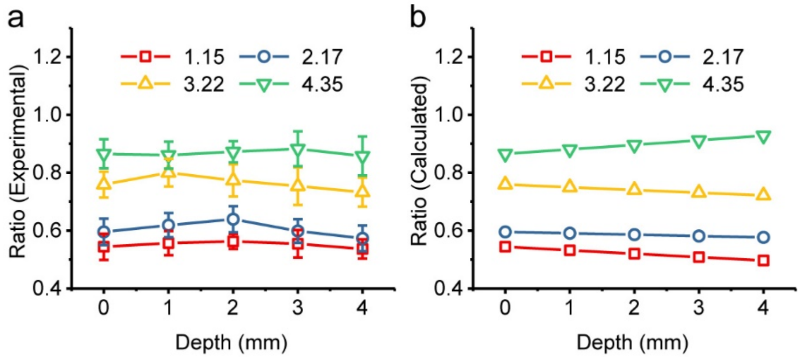

Supplementary Figure 23. The influence of attenuation coefficients on depth-dependent ratiometric signals. a, Ratiometric signals for different pH obtained from tissue phantom imaging in **Figure 6b** versus penetration depth, showing excellent reliability with increased depth. Data point with its error bar stands for mean ± s.d. derived from n = 3 replicated measurements of every pixel in the capillaries. **b**, Calculated ratiometric signals for different pH versus penetration depth based on the mathematic description in **Supplementary Note 2**, in agreement with the experiment results. We calculated the ratios at 1-4 mm depth for each pH group using **Supplementary Equation 13** based on the ratio values at 0 mm depth and $\Delta\tau$ derived from single-exponential fitting curves in **Figure 6c**. As shown in **Supplementary Table 6**, the calculated ratios show good agreement with the experiment results with low coefficient of variation (CV) for each pH group at varying depth. The results demonstrate that the key to achieve reliable ratiometric imaging in vivo is to minimize the difference of attenuation coefficients ($|\Delta\tau|$) between two signals.

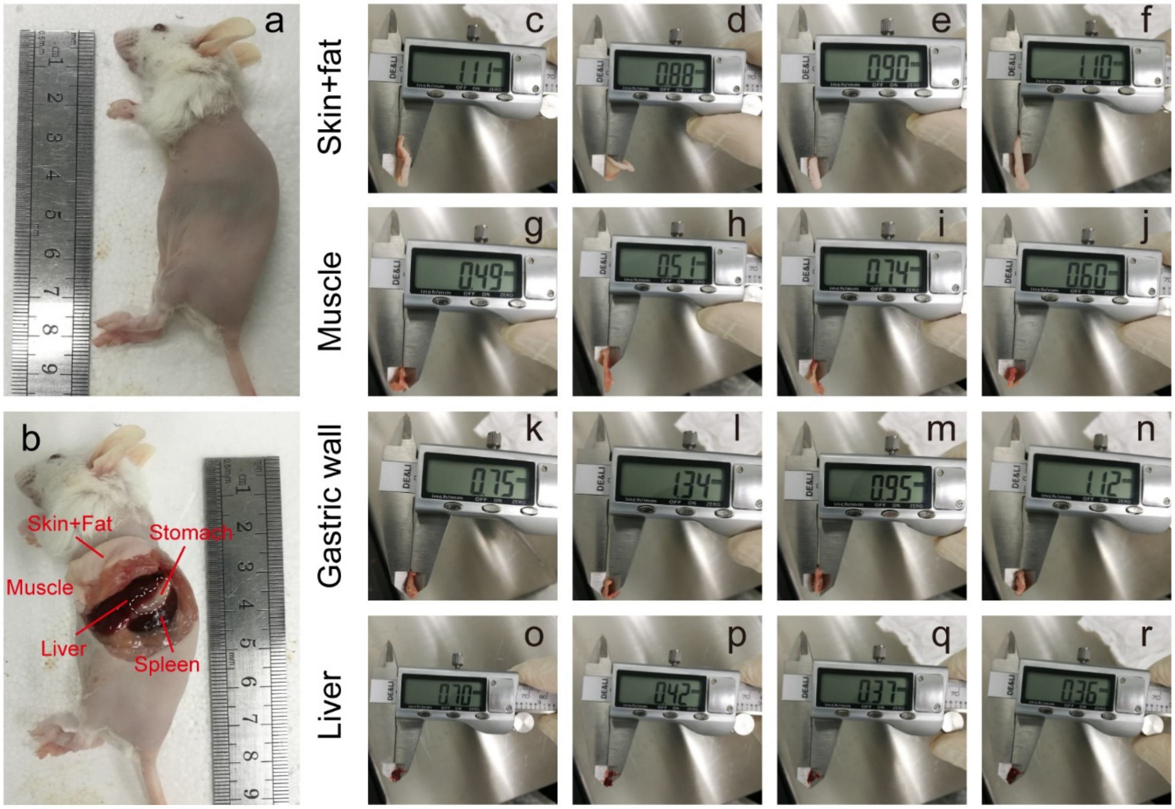

Supplementary Figure 24. Physical measurement of the thicknesses of tissue covered on mouse gastric fluid. a, **b**, Digital camera photos of a shaved ICR mouse before/after dissection, showing that the major area of stomach (white dotted circle) is covered by skin, fat and muscle tissue and only a small fraction of stomach is covered by the edge of liver. **c-r**, Repeated measurement of the tissue thickness by digital caliper at four different locations. **c-f**, The average total thickness of skin and fat is 1.00 ± 0.12 mm; **g-j**, The average thickness of muscle is 0.59 ± 0.11 mm; **k-n**, The average thickness of gastric wall is 1.04 ± 0.25 mm; **o-r**, The average thickness of liver (edge) is 0.46 ± 0.16 mm. Therefore, the tissue depth of non-invasive imaging of gastric fluid is about 2.63 ± 0.48 or 3.09 ± 0.64 (with liver) mm, within the range of 2-4 mm.

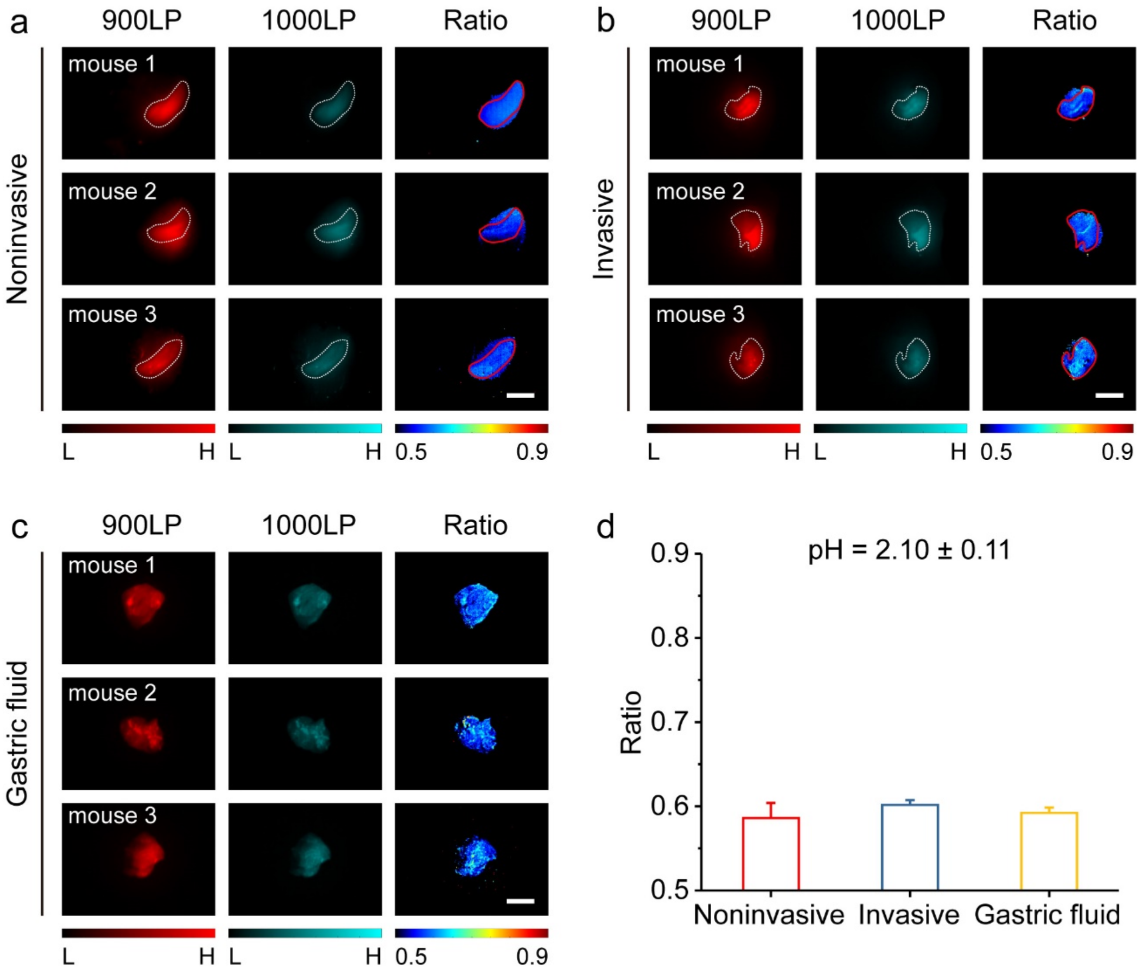

Supplementary Figure 25. In vivo ratiometric fluorescence imaging of gastric pH in low pH group. a, **b**, **c**, Fluorescence images and corresponding ratiometric images of mice stomach gavaged with 200 μL simulated gastric fluid of pH 1.3 at three imaging modes. **a**, Noninvasive imaging at ~2-4 mm tissue depth; **b**, Invasive imaging of gastric fluid covered by ~1 mm thickness of gastric wall; **c**, Imaging of exposed gastric fluid. The measurement of tissue depth/thickness is shown in **Supplementary Figure 24**. red channel: 900-1700 nm; cyan channel: 1000-1700 nm; ratio channel: F_{1000LP}/F_{900LP}. Color bar ranges from 5000 to 30000 for the red/cyan channel in a and b, 0 to 30000 for the red/cyan channel in c. The detailed imaging parameters for each image are listed in **Supplementary Table 1**. Imaging was performed independently on n = 3 biological replicates for each group with similar results. Scale bar, 5 mm. **d**, Ratiometric signals measured from the images. Data point with its error bar stands for mean ± s.d. derived from n = 3 biologically independent mice.

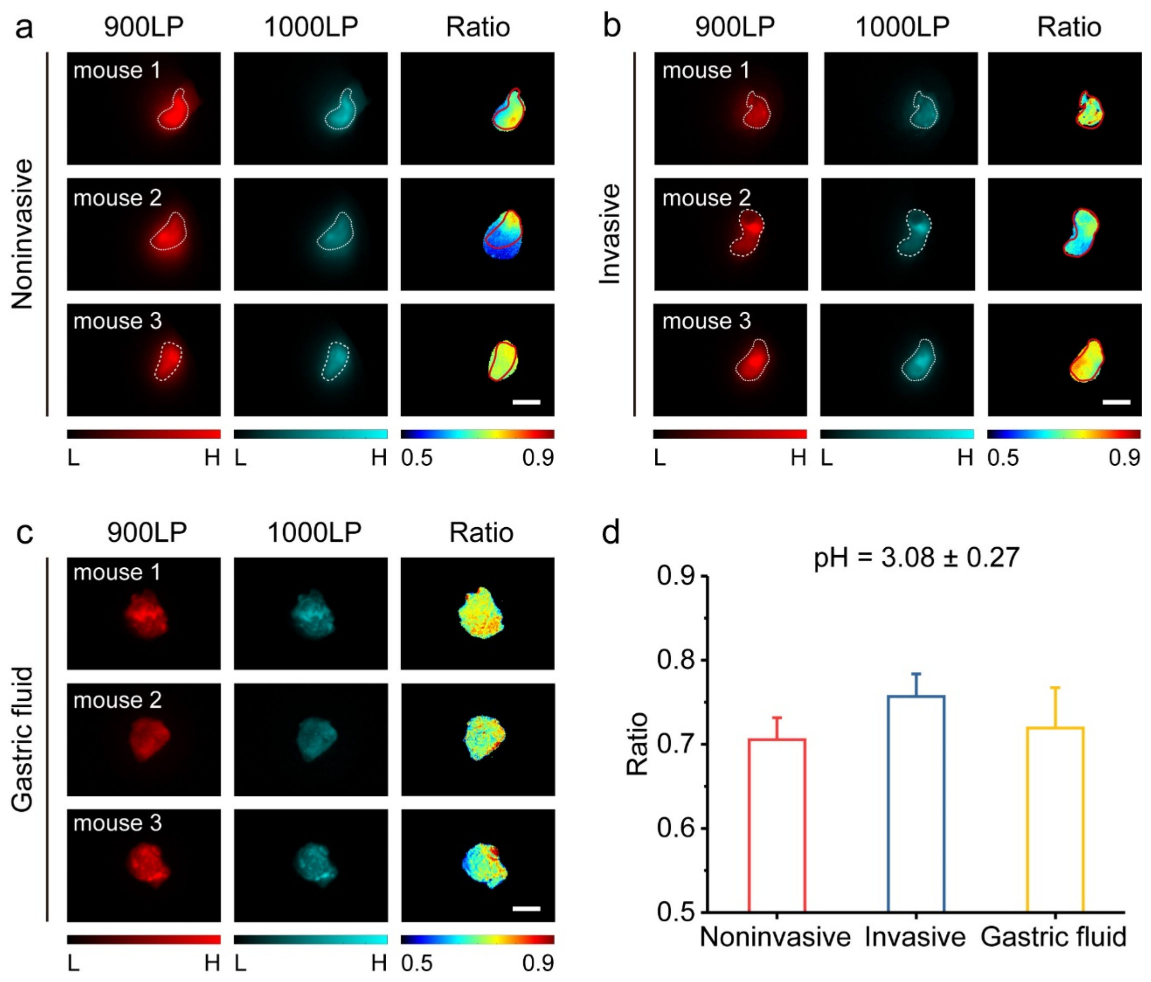

Supplementary Figure 26. In vivo ratiometric fluorescence imaging of gastric pH in high pH group. a, **b**, **c**, Fluorescence images and corresponding ratiometric images of mice stomach gavaged with 200 μL simulated gastric fluid of pH 2.5 at three imaging modes. **a**, Noninvasive imaging at ~2-4 mm tissue depth; **b**, Invasive imaging of gastric fluid covered by ~1 mm thickness of gastric wall; **c**, Imaging of exposed gastric fluid. The measurement of tissue depth/thickness is shown in **Supplementary Figure 24**. red channel: 900-1700 nm; cyan channel: 1000-1700 nm; ratio channel: F_{1000LP}/F_{900LP}. Color bar ranges from 5000 to 30000 for the red/cyan channel in a and b, 0 to 30000 for the red/cyan channel in c. The detailed imaging parameters for each image are listed in **Supplementary Table 1**. Imaging was performed independently on n = 3 biological replicates for each group with similar results. Scale bar, 5 mm. **d**, Ratiometric signals measured from the images. Data point with its error bar stands for mean ± s.d. derived from n = 3 biologically independent mice.

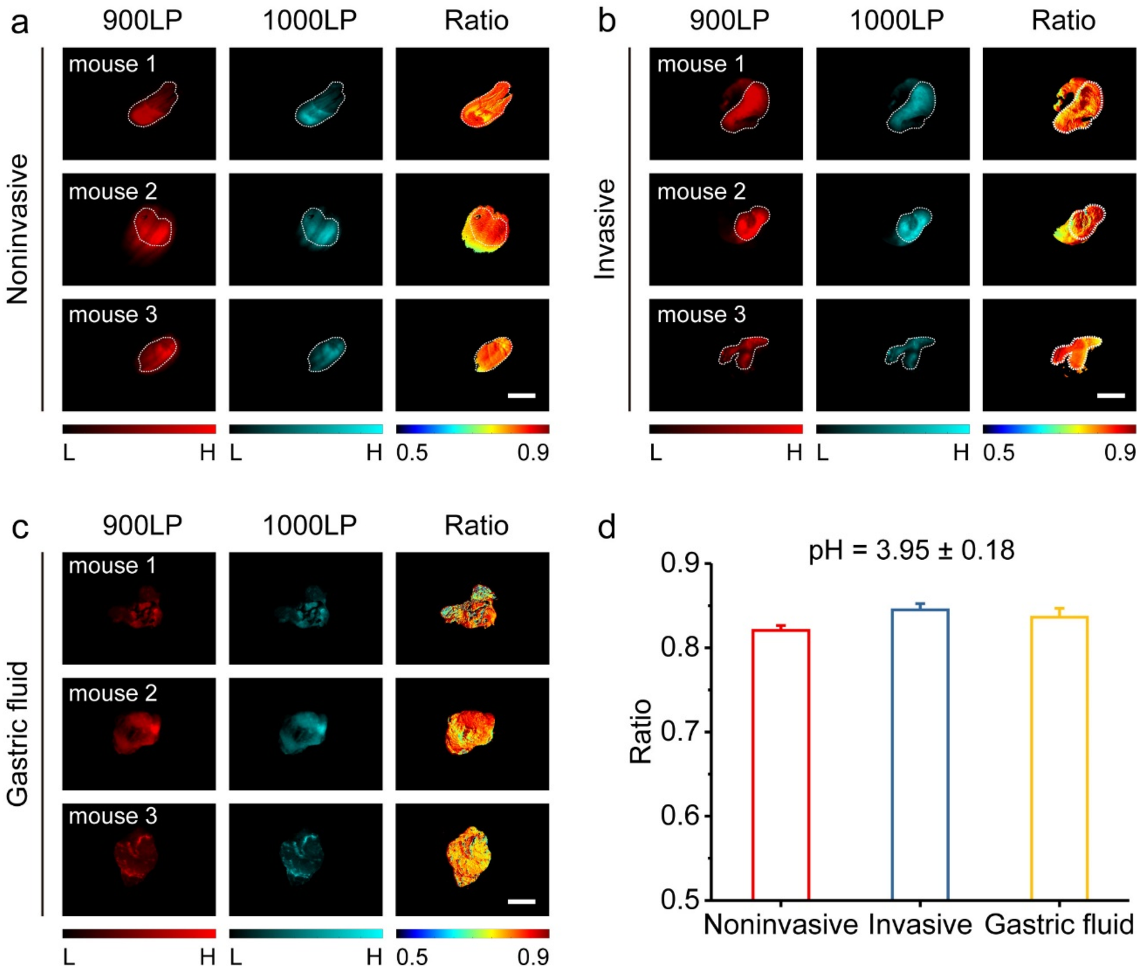

Supplementary Figure 27. In vivo ratiometric fluorescence imaging of gastric pH in normal pH group. a, **b**, **c**, Fluorescence images and corresponding ratiometric images of normal mice stomach gavaged with 20 μL BTC1070 micelle solution (dye concentration: 500 μM; solvent: deionized water) at three imaging modes. **a**, Noninvasive imaging at ~2-4 mm tissue depth; **b**, Invasive imaging of gastric fluid covered by ~1 mm thickness of gastric wall; **c**, Imaging of exposed gastric fluid. The measurement of tissue depth/thickness is shown in **Supplementary Figure 24**. red channel: 900-1700 nm; cyan channel: 1000-1700 nm; ratio channel: F_{1000LP}/F_{900LP}. Color bar ranges from 5000 to 30000 for the red/cyan channel in a and b, 0 to 30000 for the red/cyan channel in c. The detailed imaging parameters for each image are listed in **Supplementary Table 1**. Imaging was performed independently on n = 3 biological replicates for each group with similar results. Scale bar, 5 mm. **d**, Ratiometric signals measured from the images. Data point with its error bar stands for mean ± s.d. derived from n = 3 biologically independent mice.

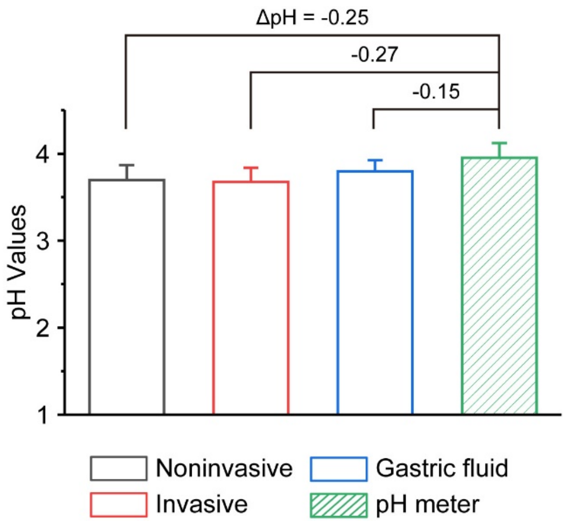

Supplementary Figure 28. Comparison of normal gastric pH measured by ratiometric imaging and pH meter. Ratio values were converted to pH values by means of the calibration functions at corresponding depth (**Supplementary Note 3**). ΔpH = average pH resolved from ratometric imaging **-** average pH measured by standard pH meter. Detailed pH data were summarised in **Supplementary Table 7**. Data point with its error bar stands for mean ± s.d. derived from n = 3 biologically independent mice.

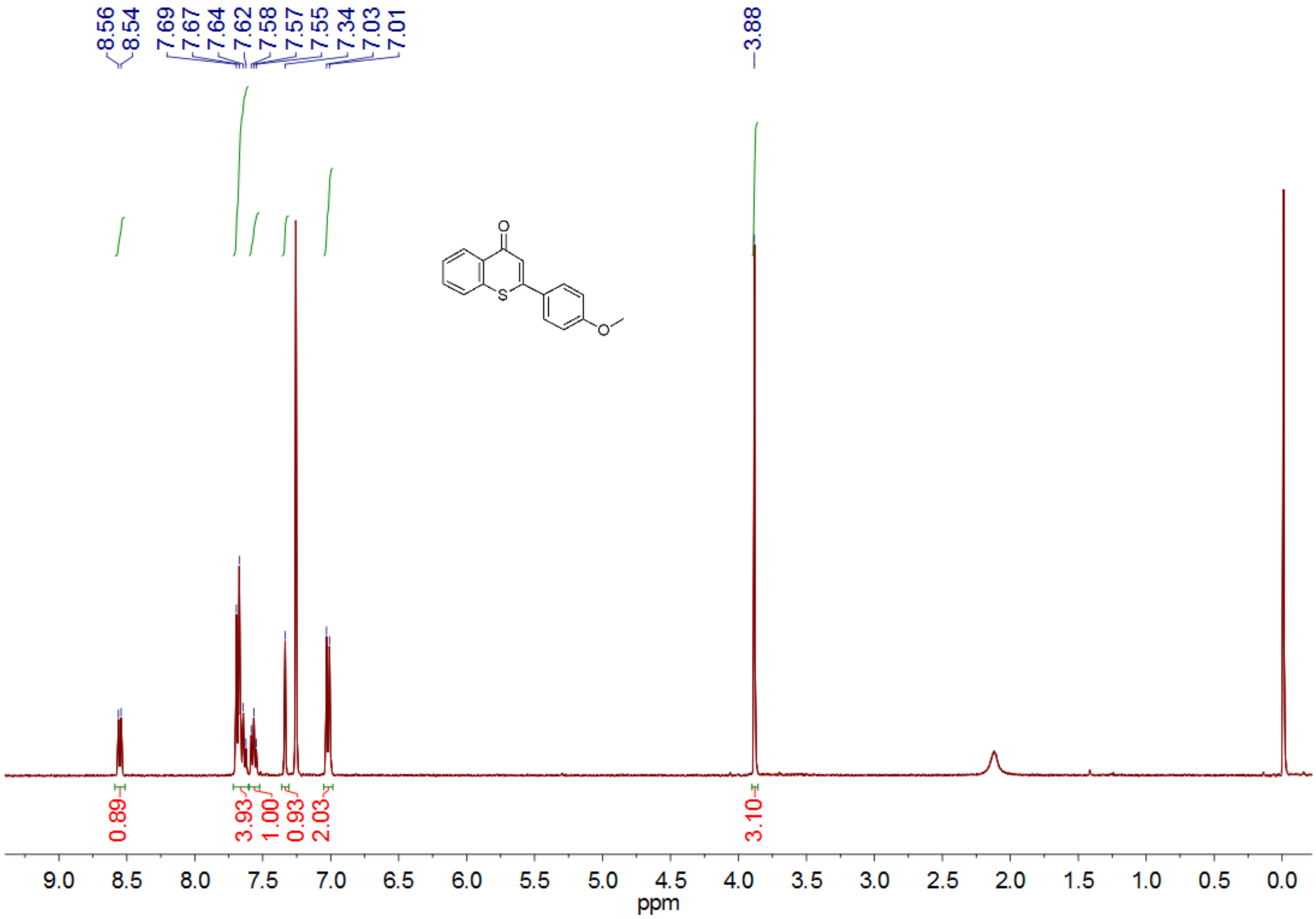

Supplementary Figure 29. ^{1}H-NMR spectrum of compound 1b in $CDCl_3$.

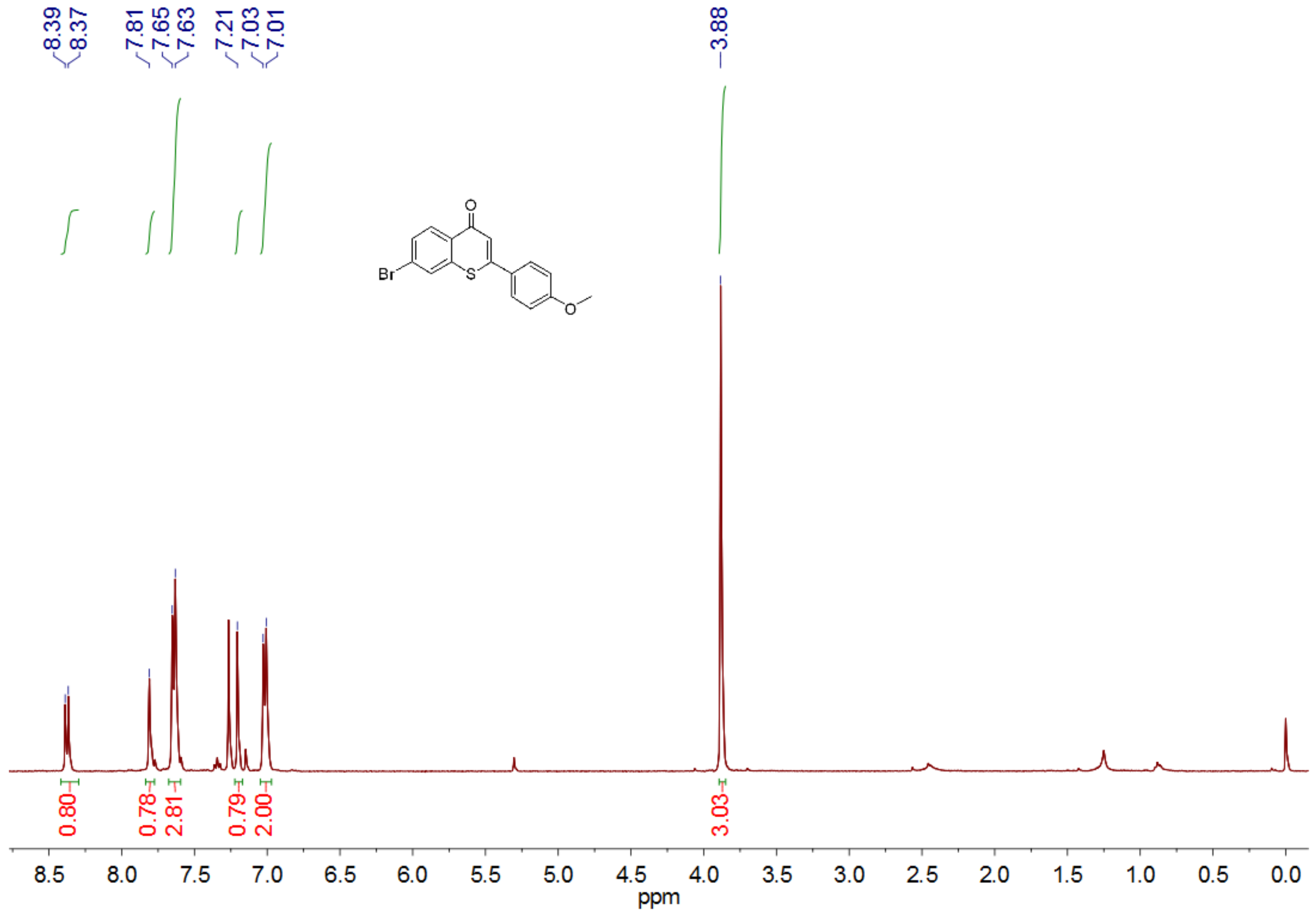

Supplementary Figure 30. ^{1}H-NMR spectrum of compound 2b in $CDCl_3$.

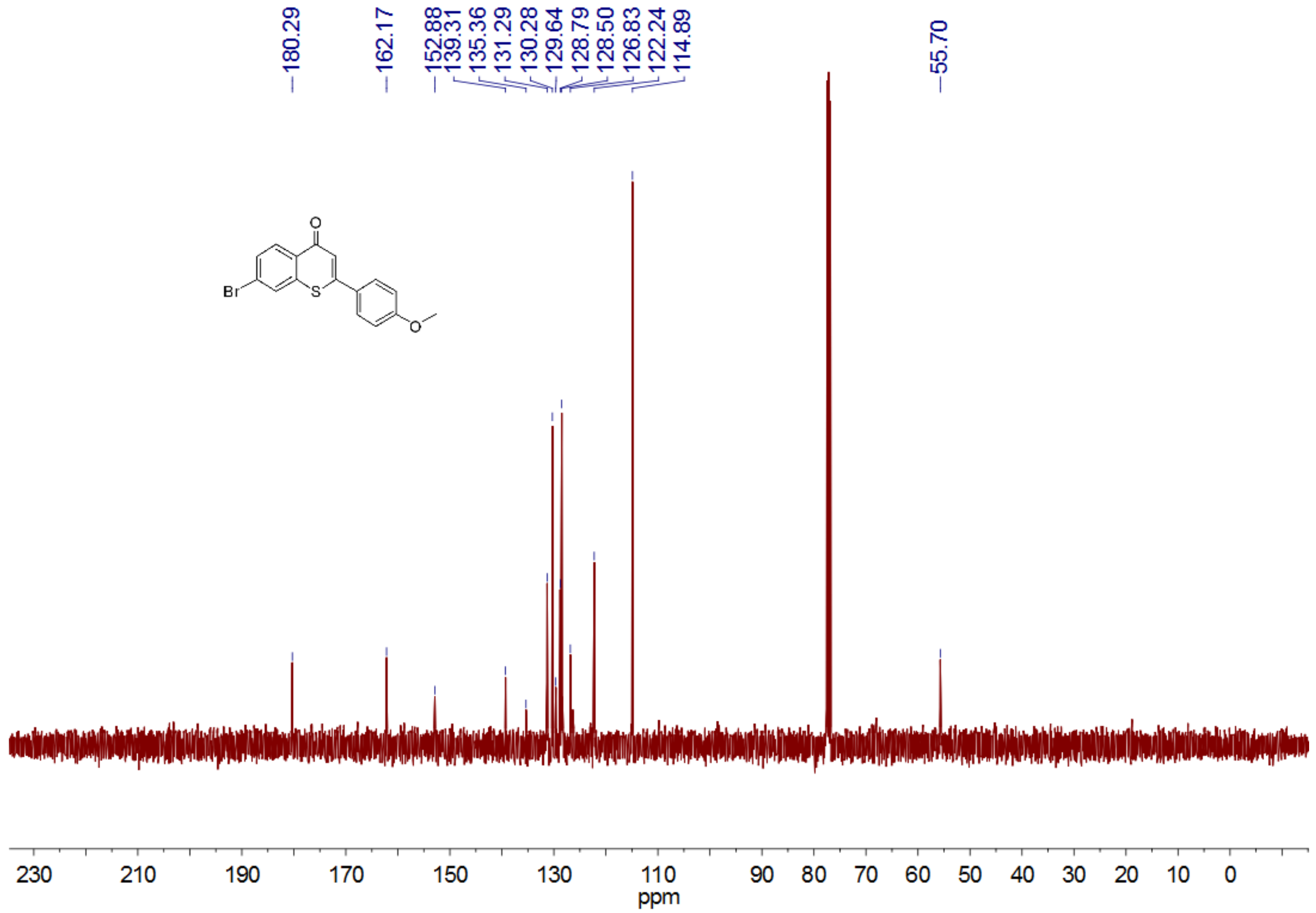

Supplementary Figure 31. **^{13}C-NMR spectrum of compound 2b in $CDCl_3$.**

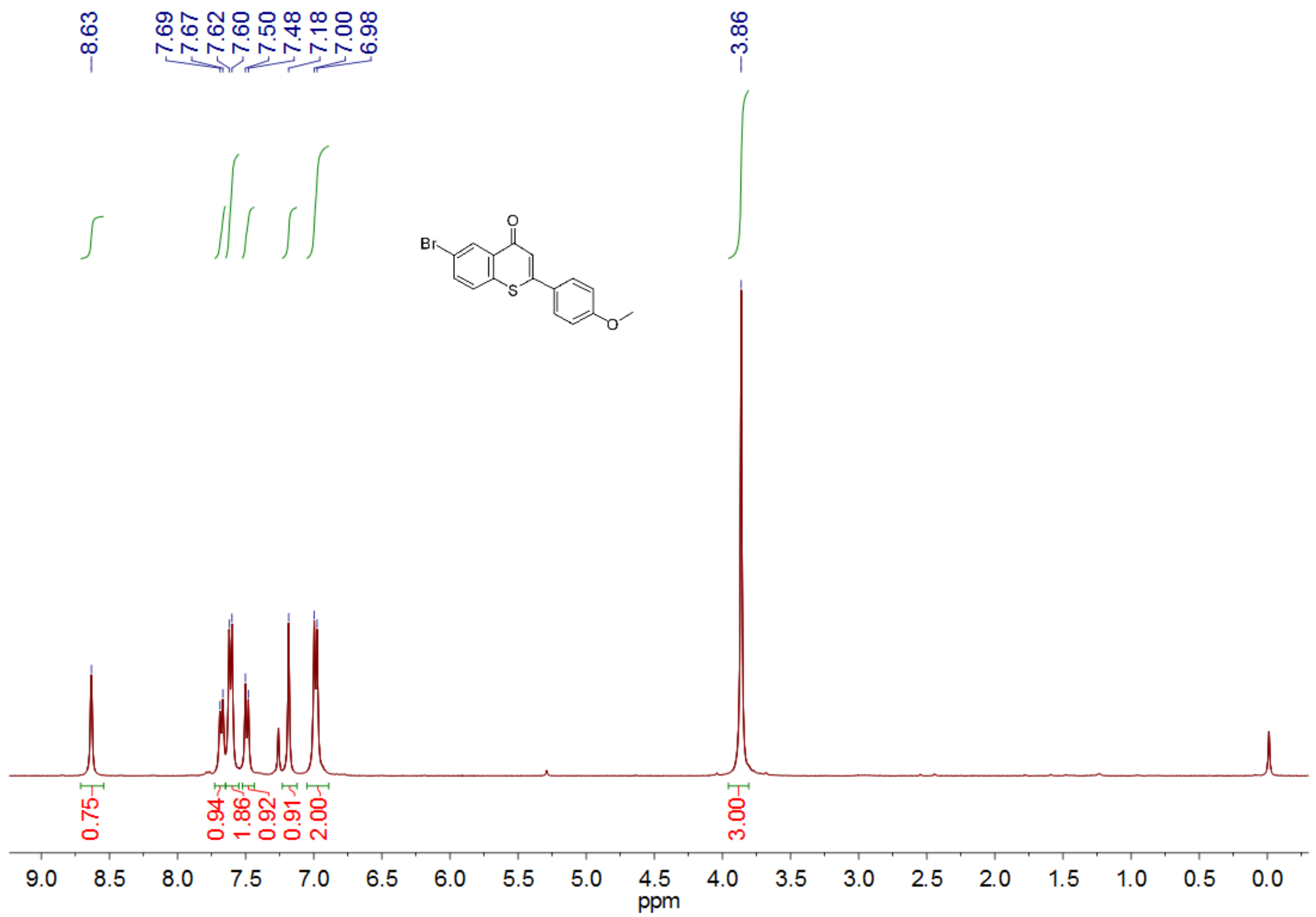

Supplementary Figure 32. ^{1}H-NMR spectrum of compound 3b in $CDCl_3$.

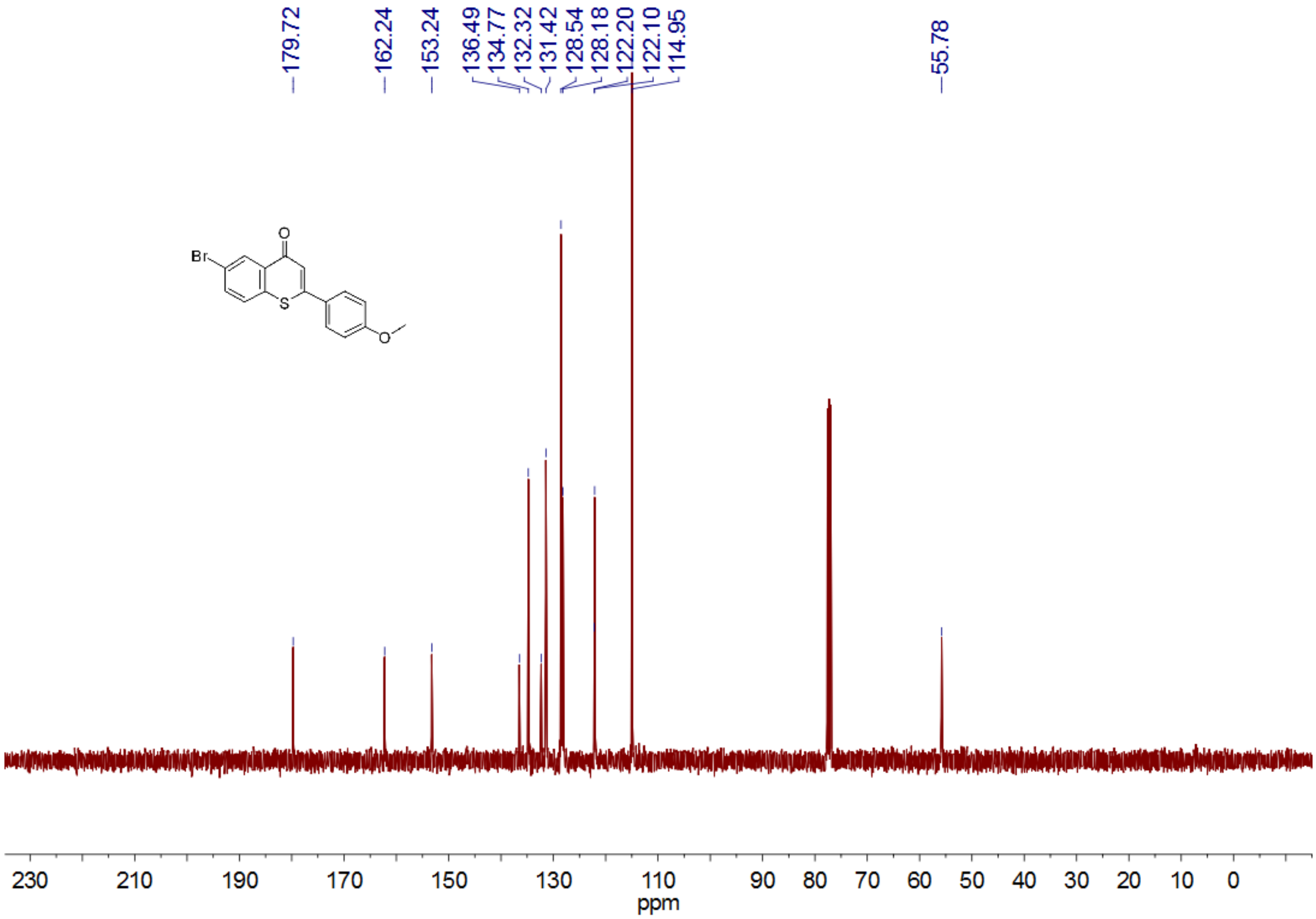

Supplementary Figure 33. ^{13}C-NMR spectrum of compound 3b in $CDCl_3$.

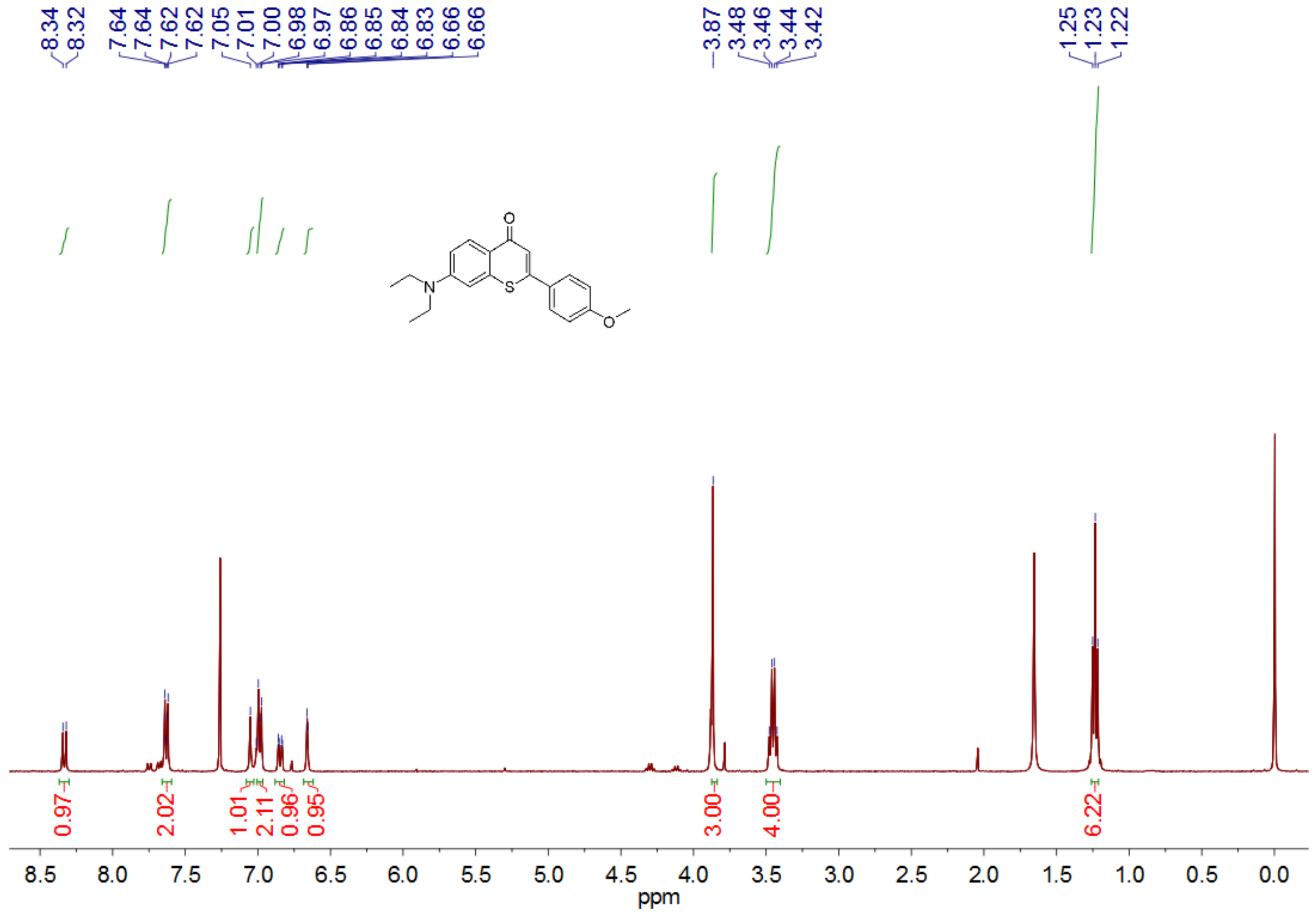

Supplementary Figure 34. ^{1}H-NMR spectrum of compound 2c in $CDCl_3$.

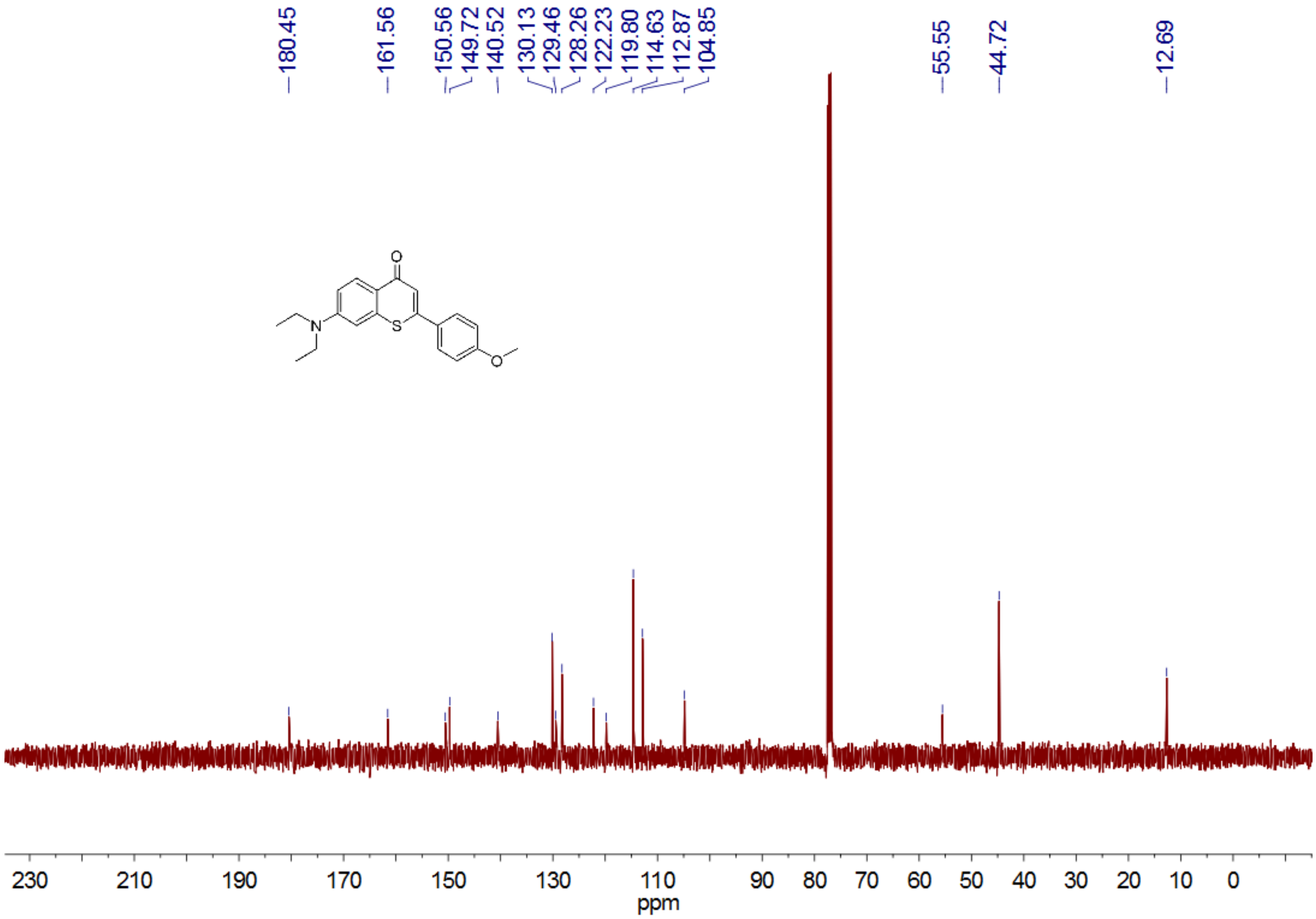

Supplementary Figure 35. ^{13}C-NMR spectrum of compound 2c in $CDCl_3$.

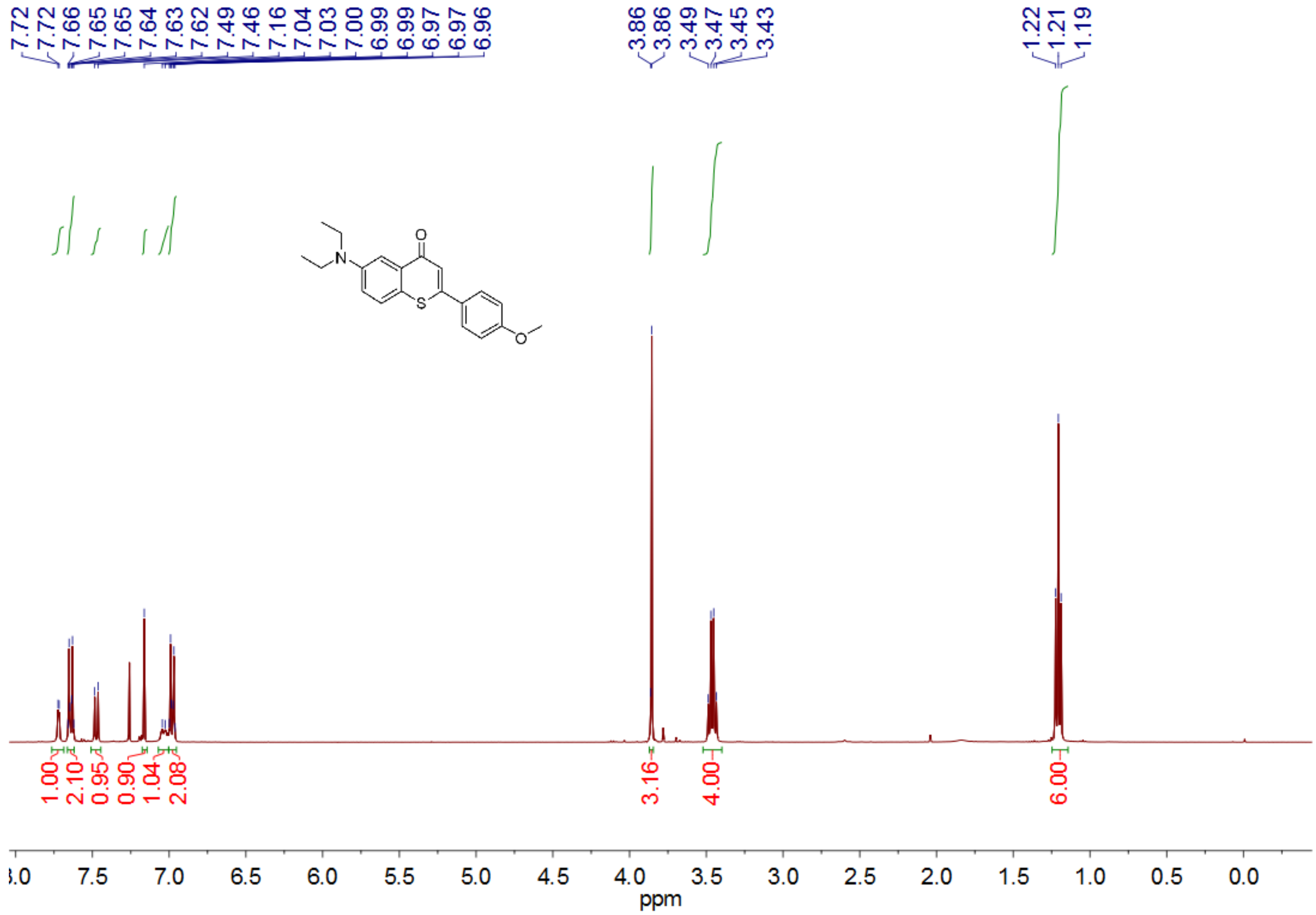

Supplementary Figure 36. ^{1}H-NMR spectrum of compound 3c in $CDCl_3$.

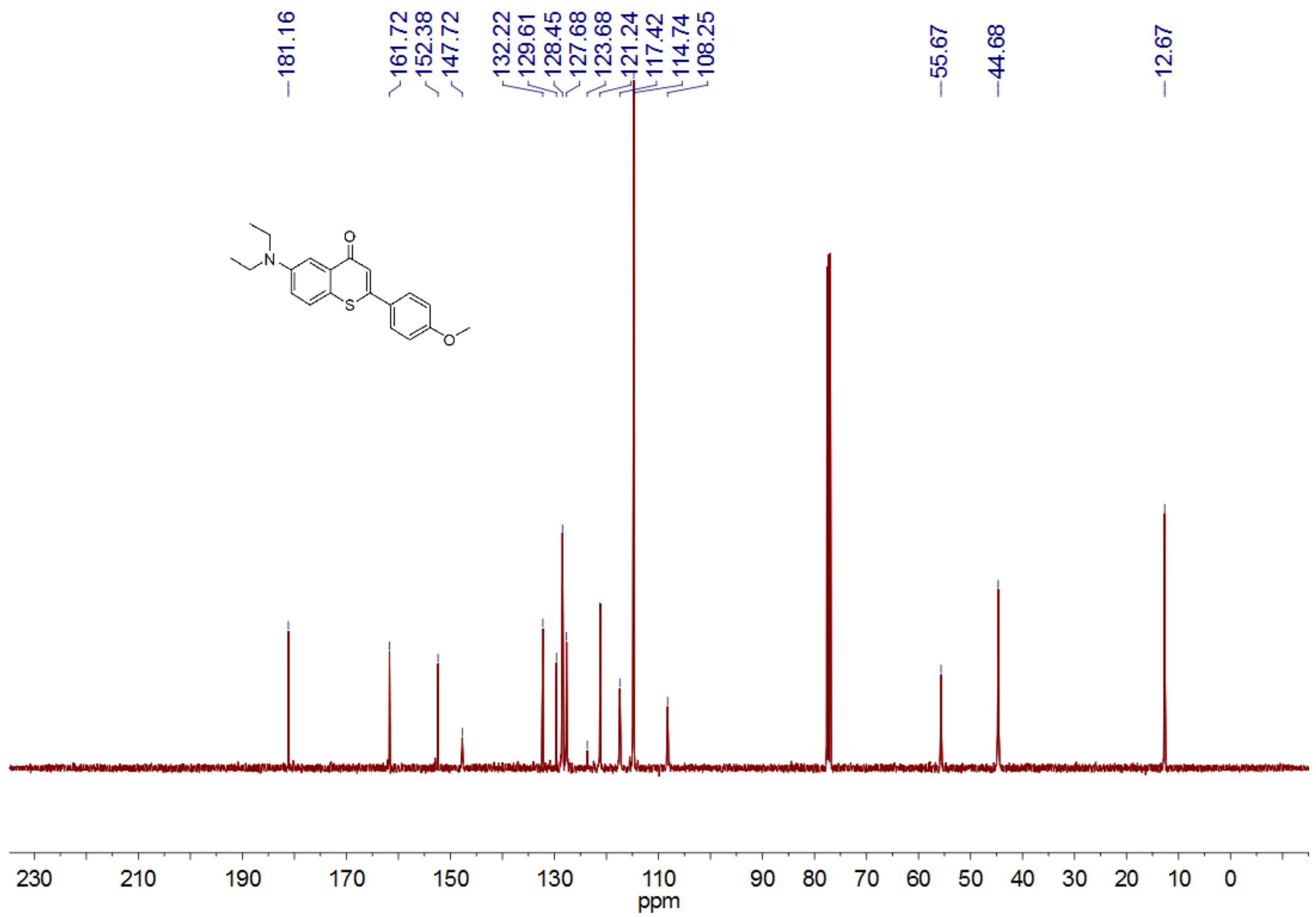

Supplementary Figure 37. ^{13}C-NMR spectrum of compound 3c in $CDCl_3$.

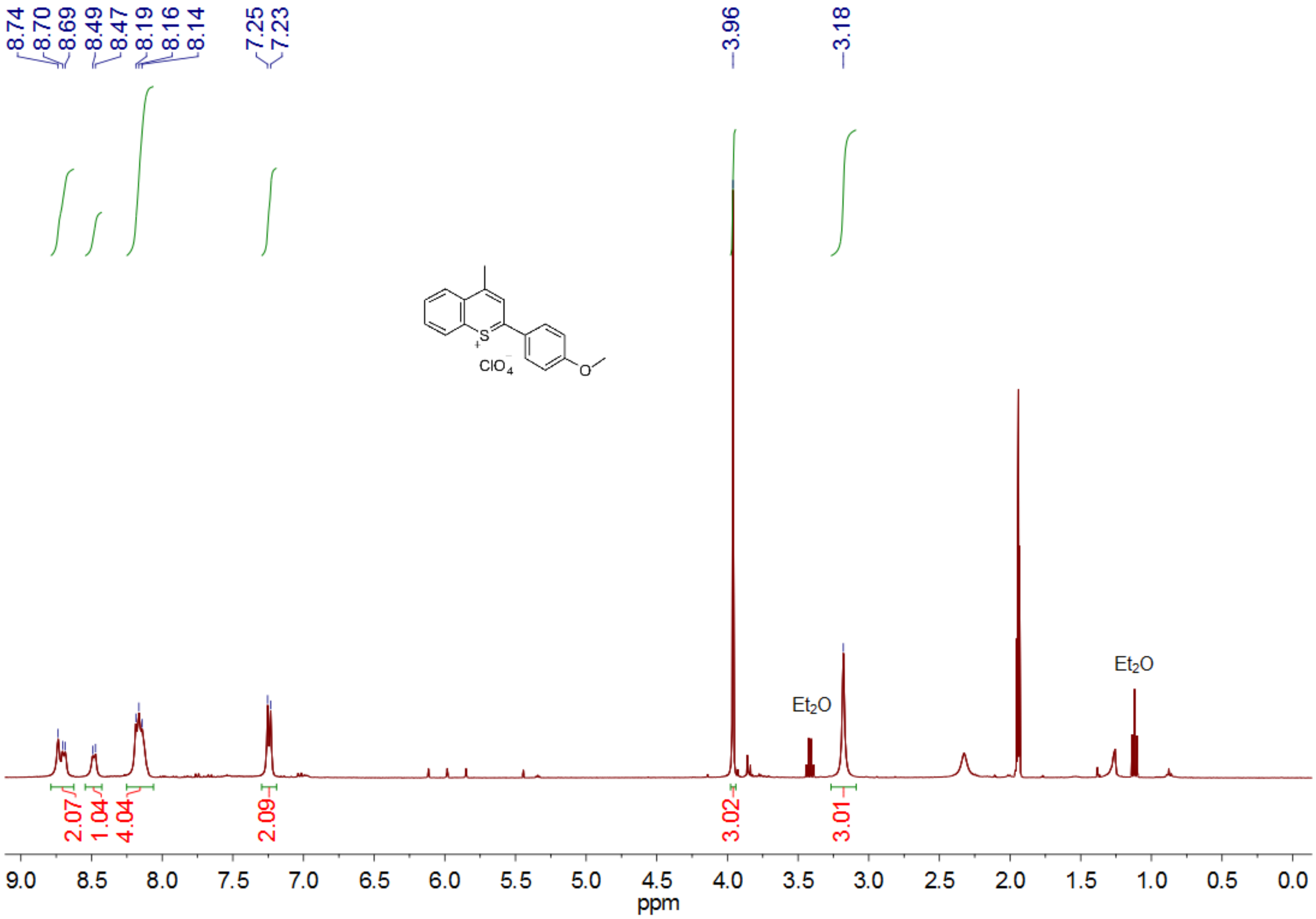

Supplementary Figure 38. ^{1}H-NMR spectrum of compound 1d in CD_3CN.

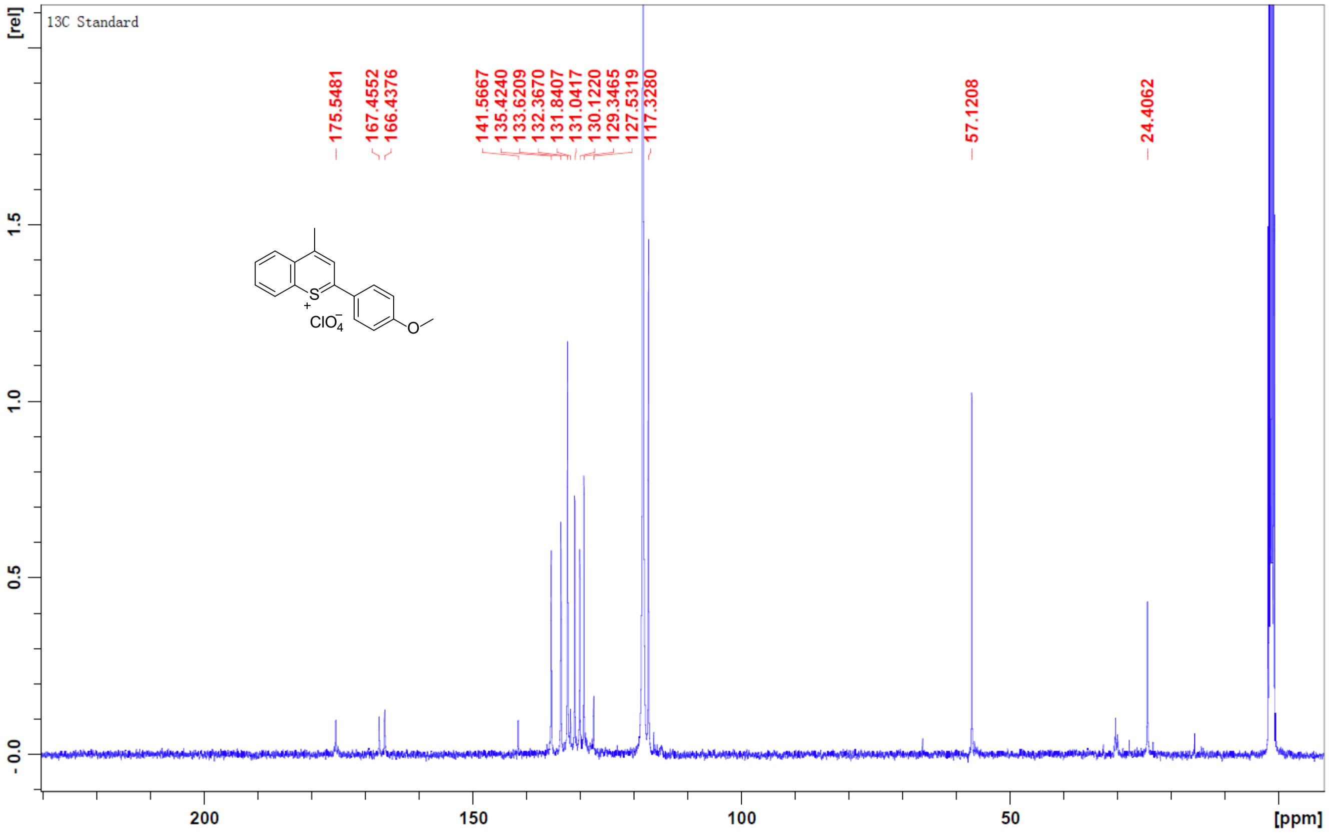

Supplementary Figure 39. ^{13}**C-NMR spectrum of compound 1d in** CD_3CN**.**

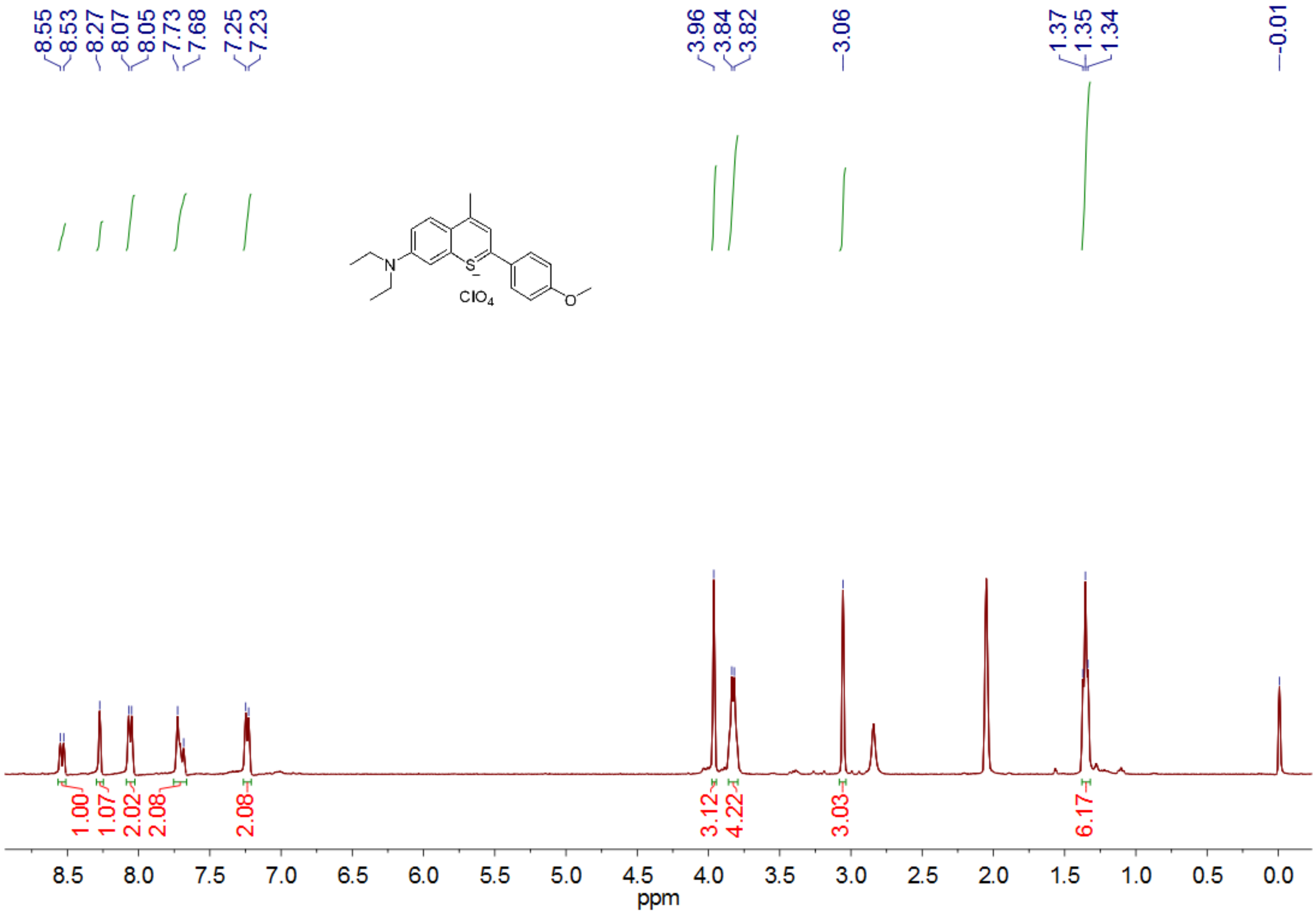

Supplementary Figure 40. ^{1}H-NMR spectrum of compound 2d in acetone-D6.

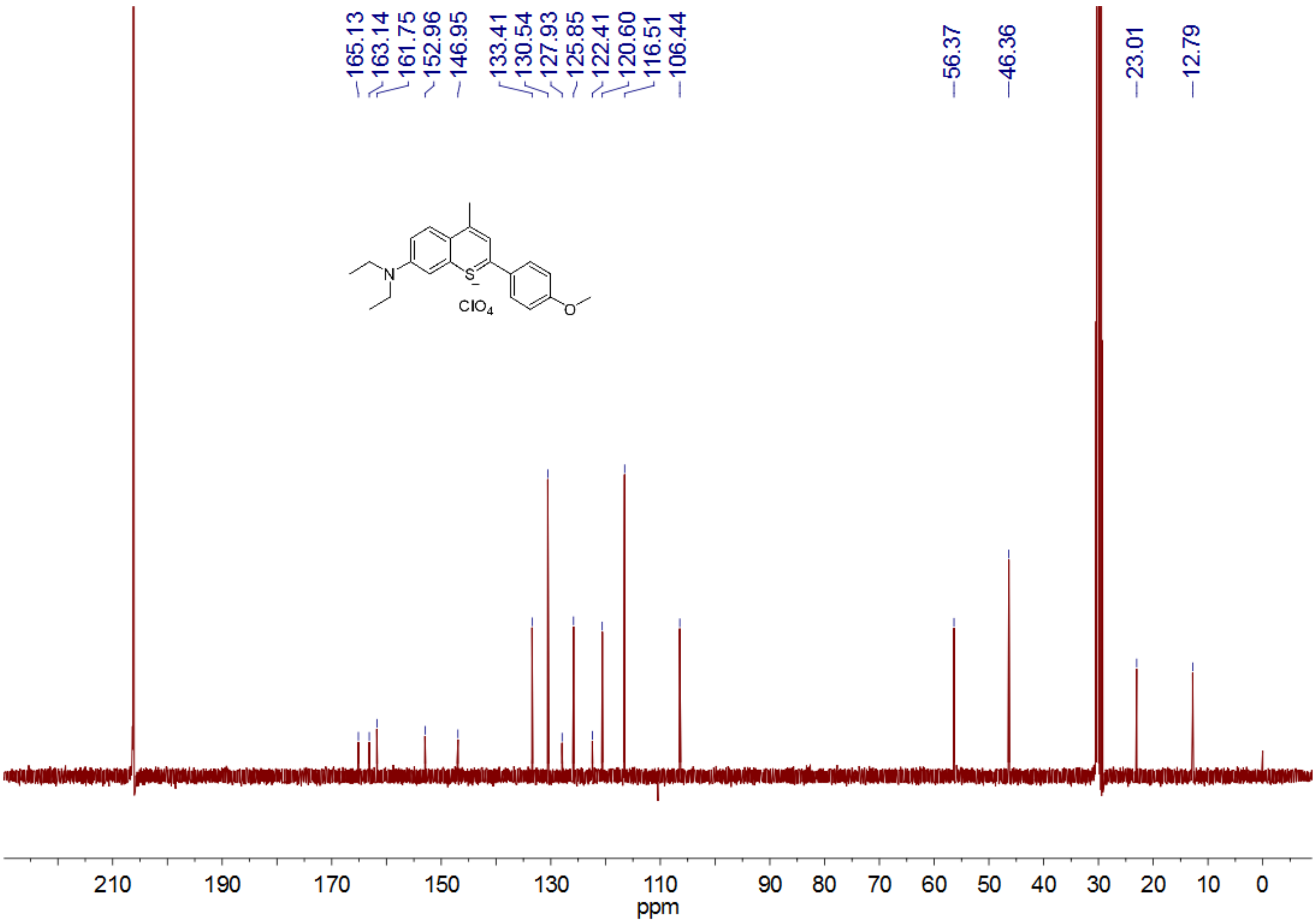

Supplementary Figure 41. ^{13}**C-NMR spectrum of compound 2d in acetone-D6.**

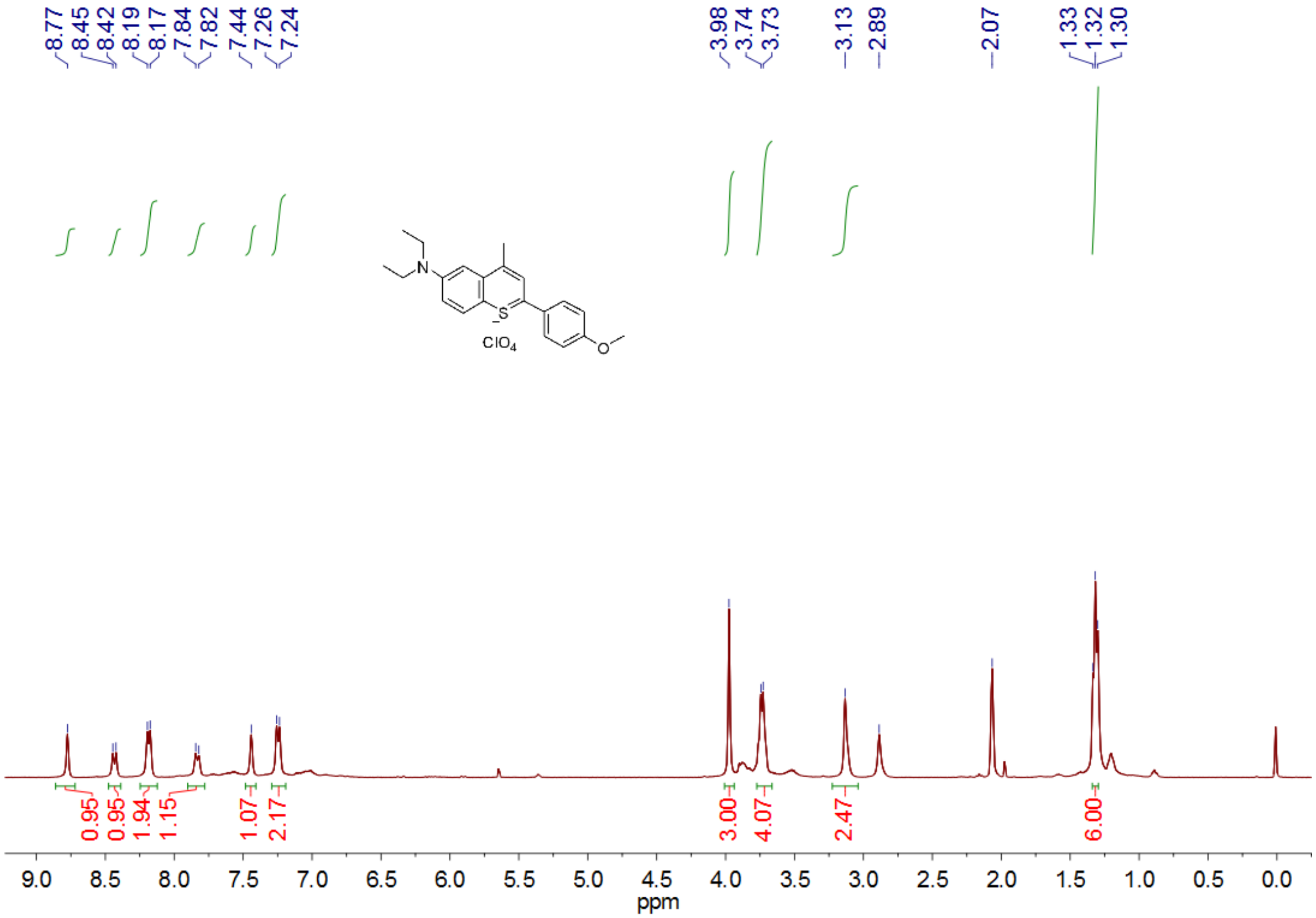

Supplementary Figure 42. ^{1}H-NMR spectrum of compound 3d in acetone-D6.

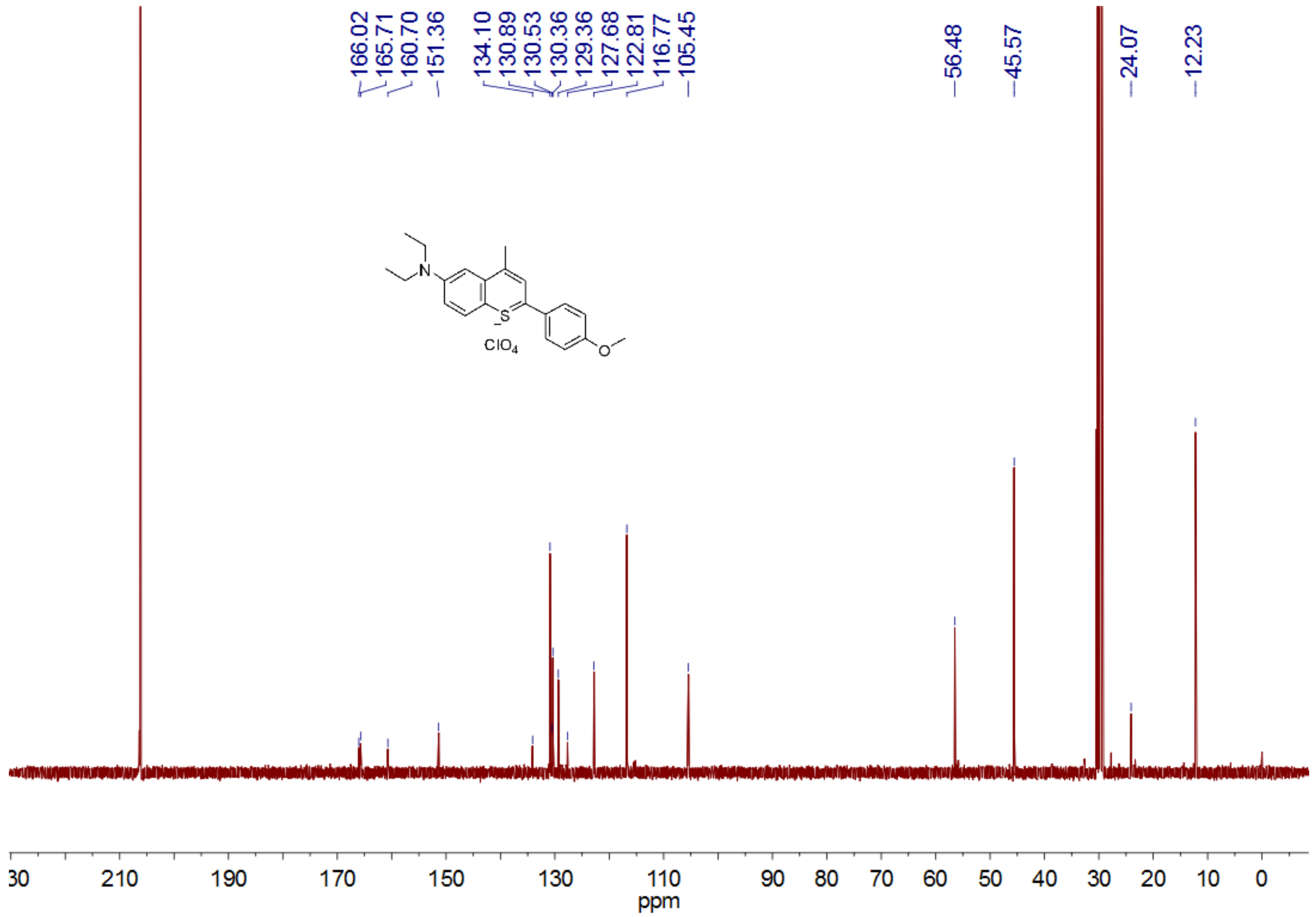

Supplementary Figure 43. ^{13}C-NMR spectrum of compound 3d in acetone-D6.

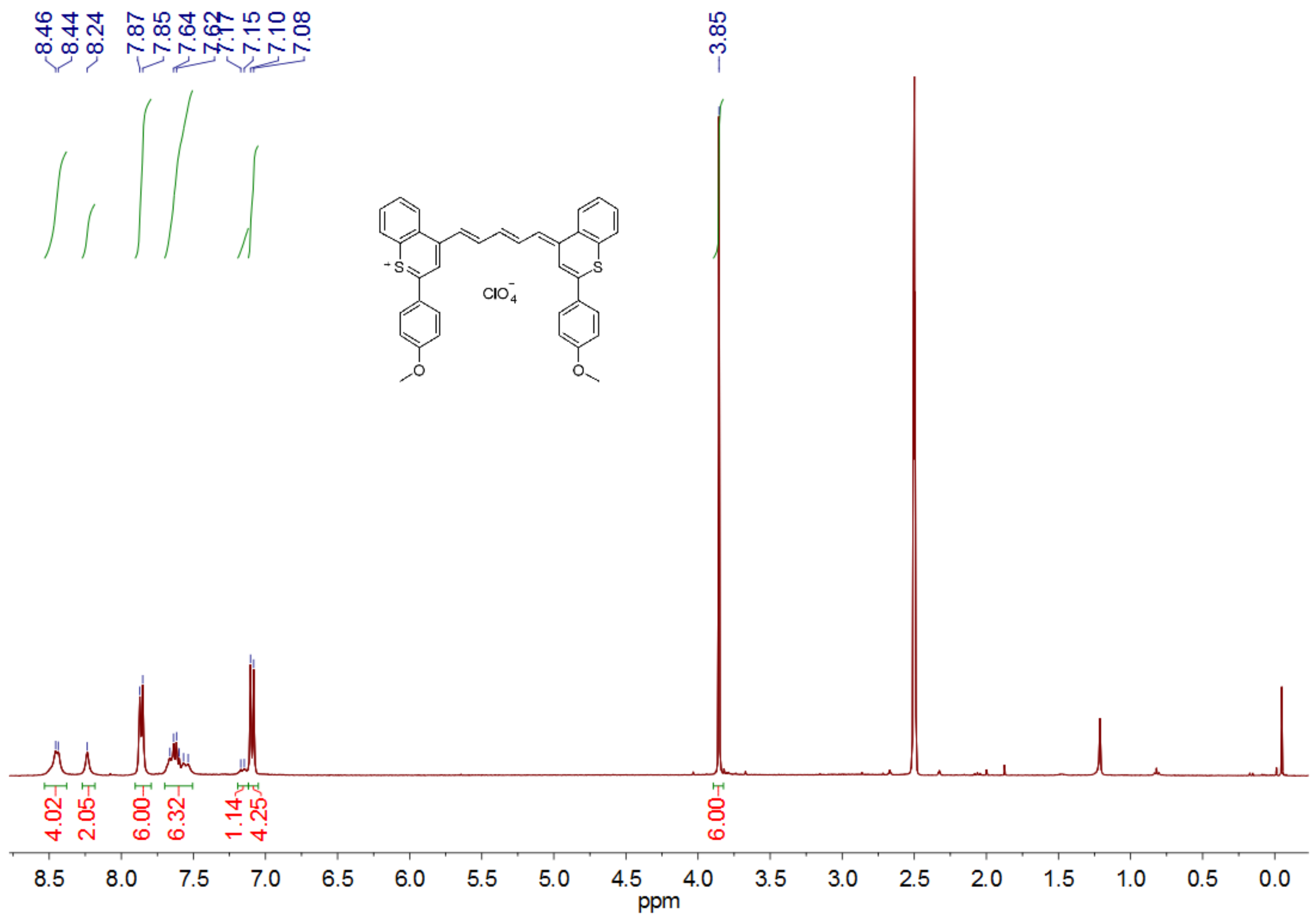

Supplementary Figure 44. ^{1}H-NMR spectrum of BTC980 in DMSO-D6.

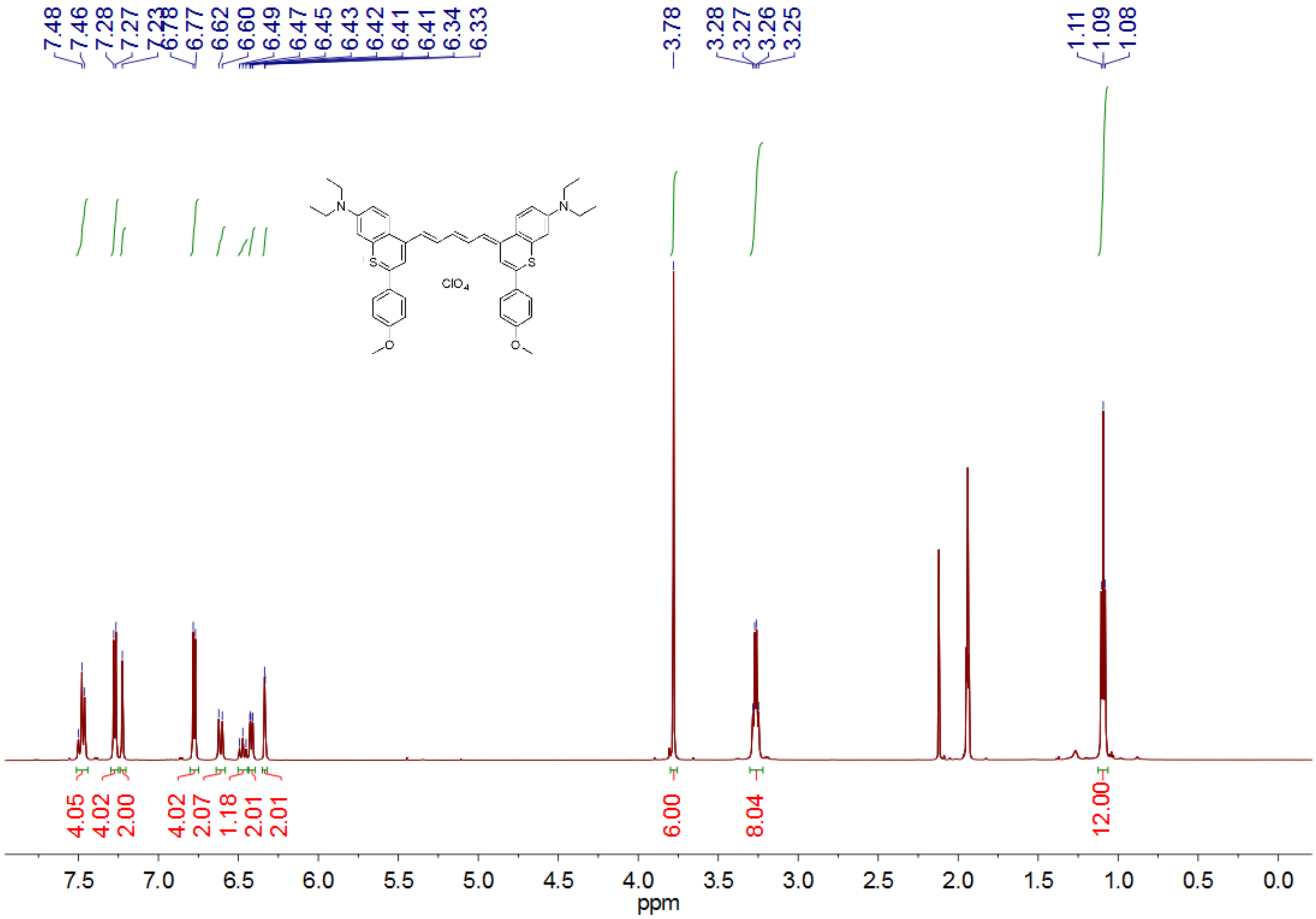

Supplementary Figure 45. ^{1}H-NMR spectrum of BTC982 in CD_3CN.

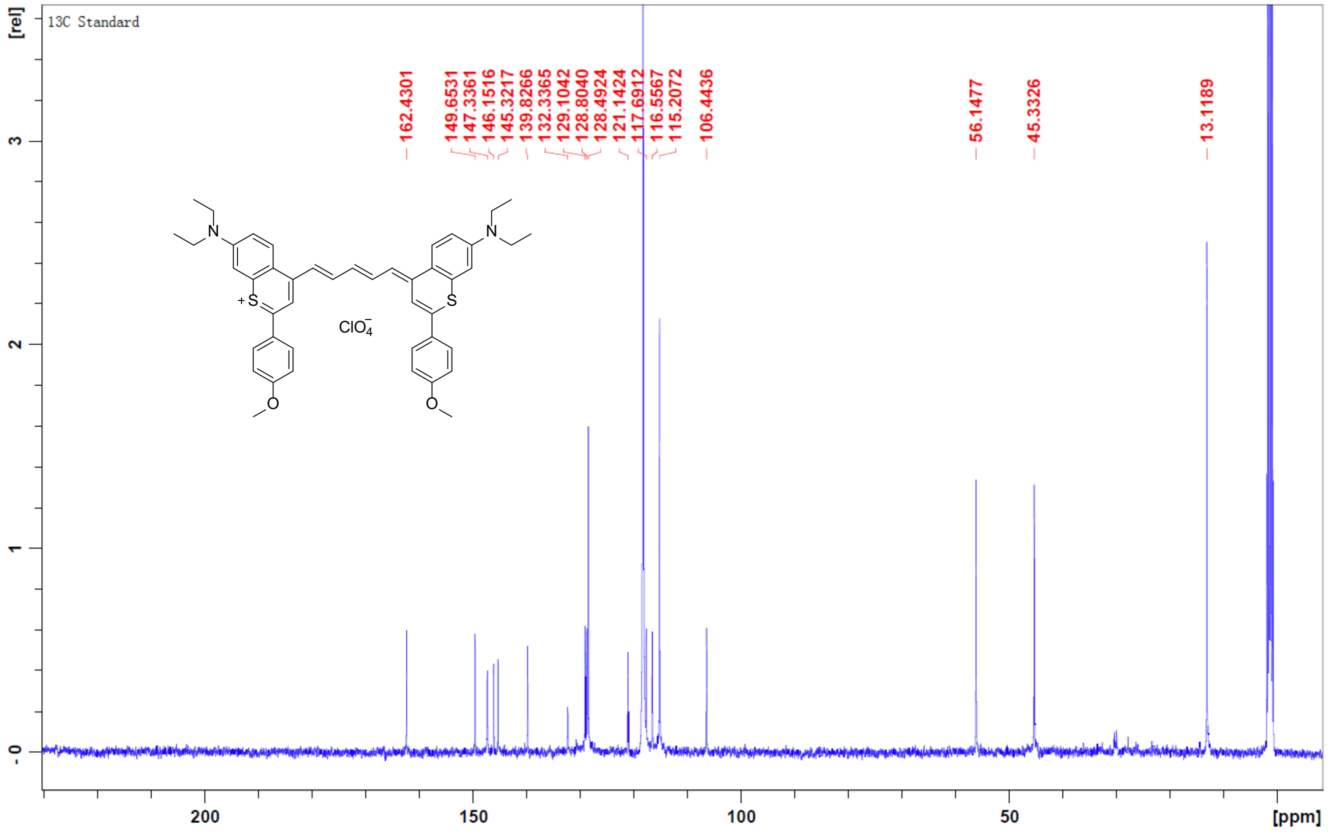

Supplementary Figure 46. **^{13}C-NMR spectrum of BTC982 in CD_3CN.**

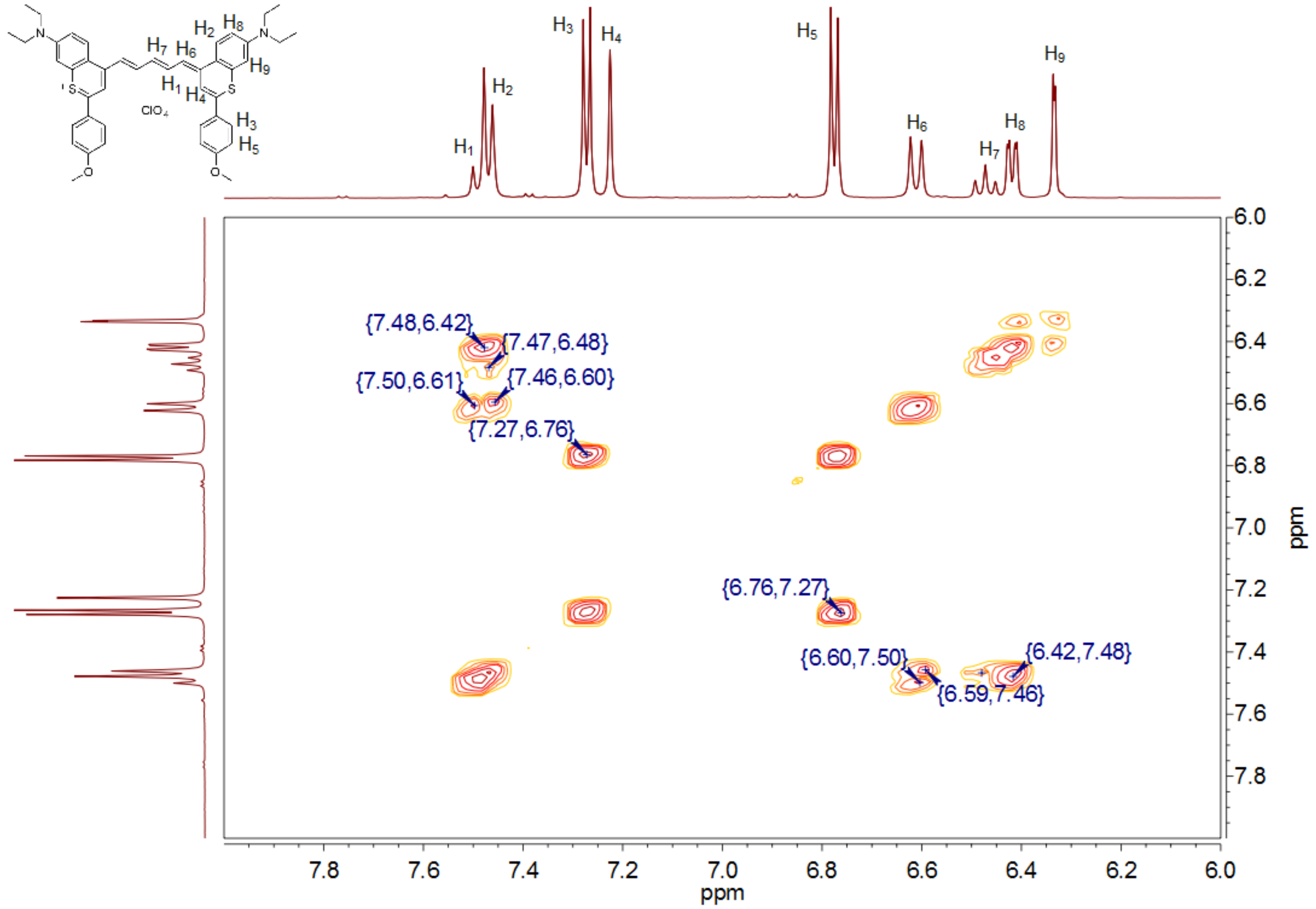

Supplementary Figure 47. ^{1}H-^{1}H COSY spectrum of BTC982 in CD_3CN.

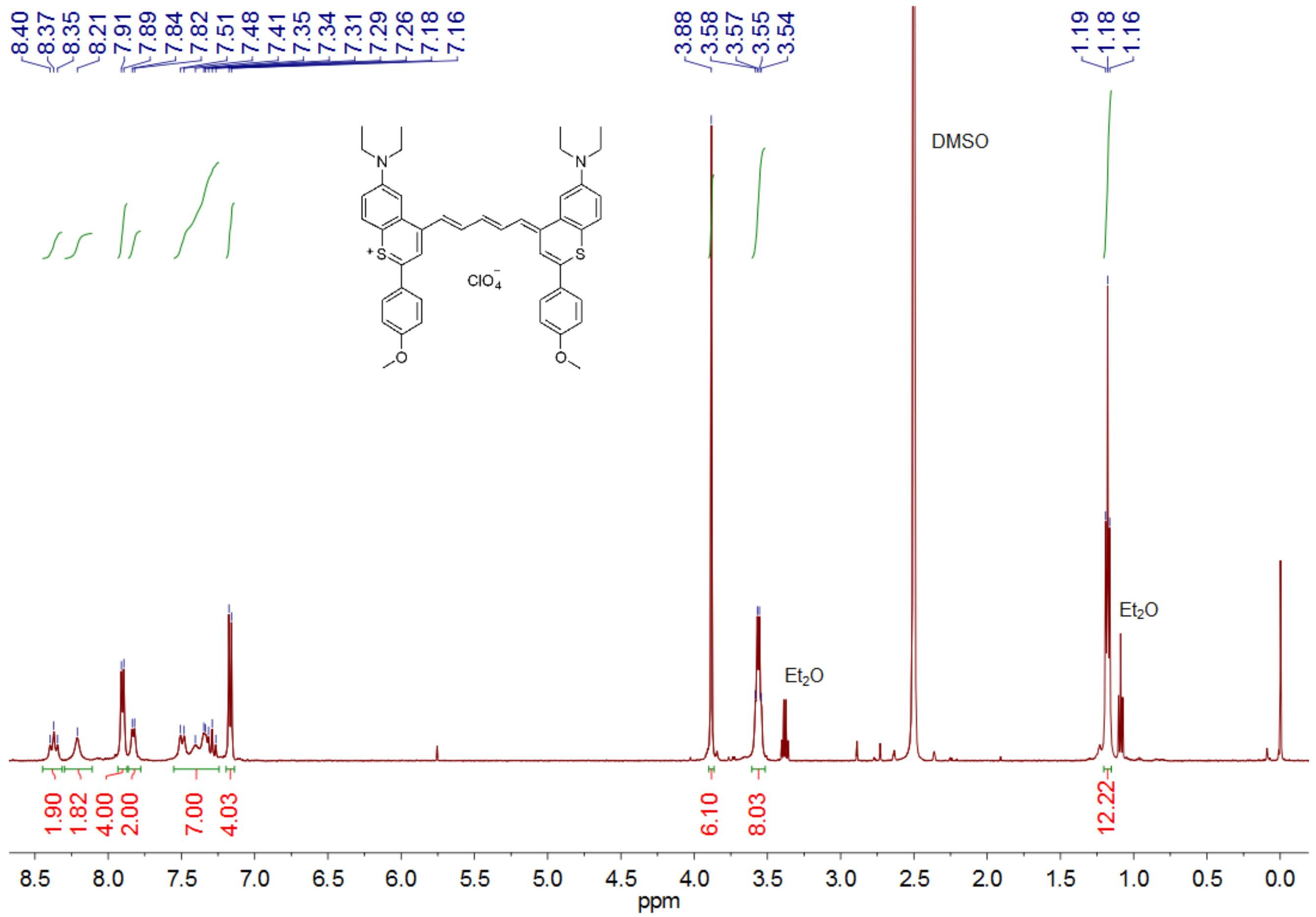

Supplementary Figure 48. ^{1}**H-NMR spectrum of BTC1070 in DMSO-D6.**

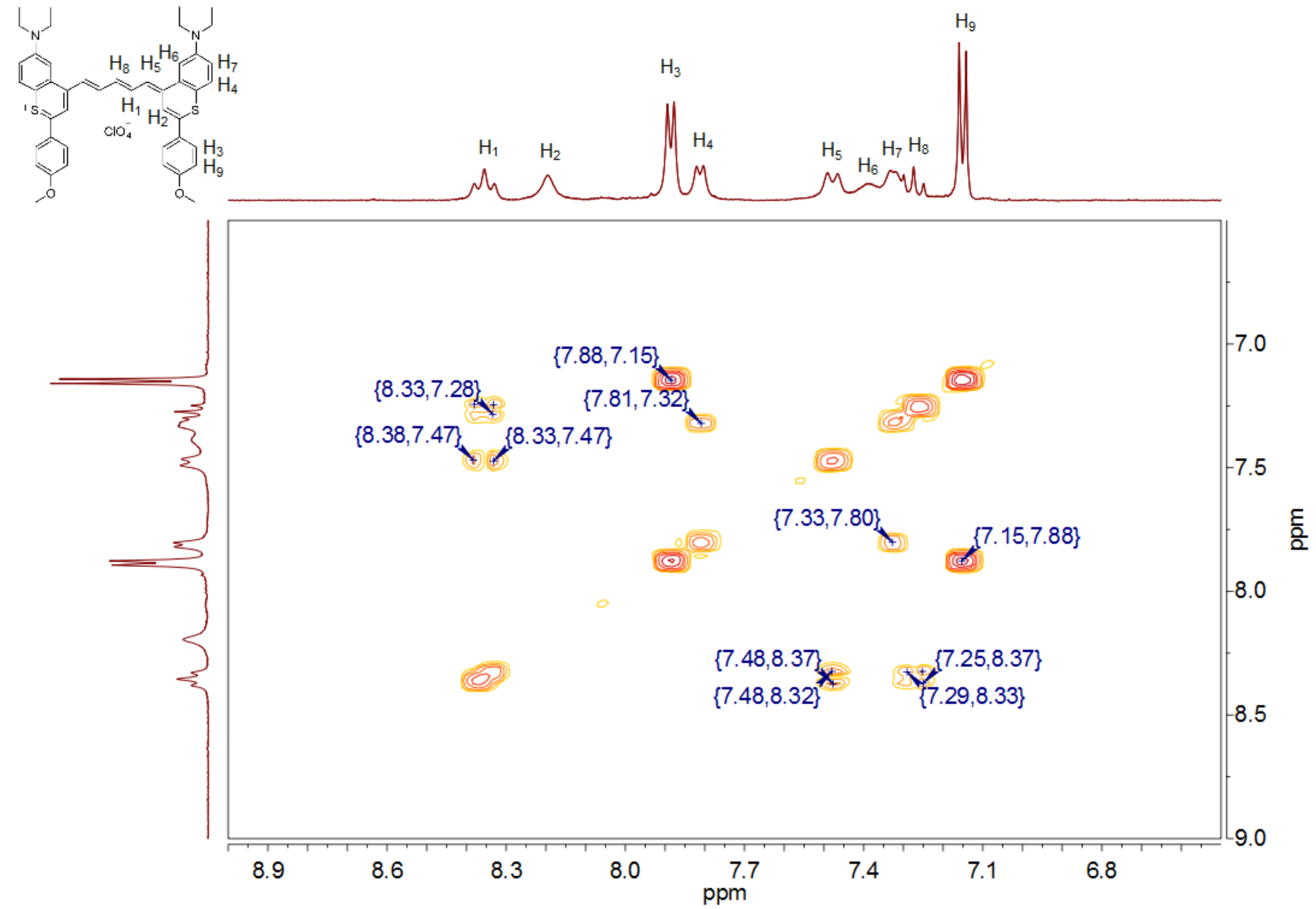

Supplementary Figure 49. ^{1}H-^{1}H COSY spectrum of BTC1070 in DMSO-D6.

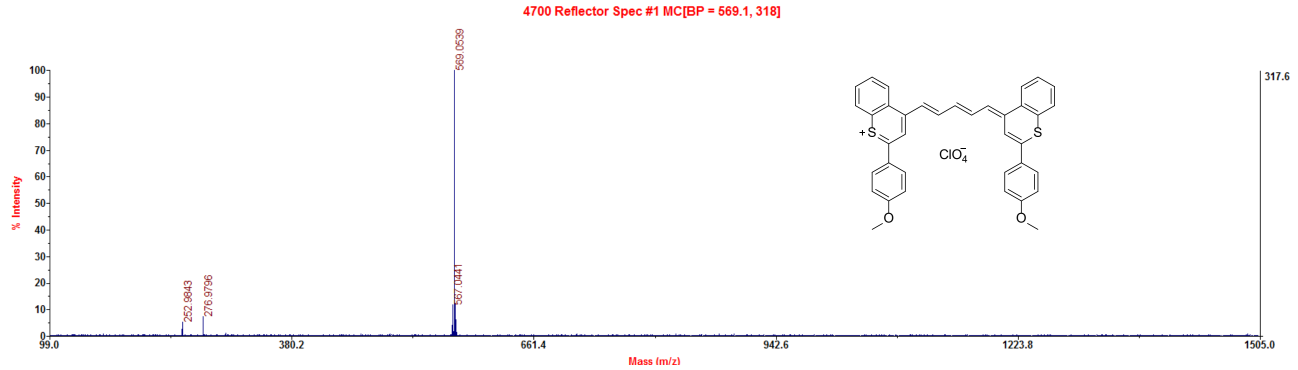

Supplementary Figure 50. Maldi-Tof/Tof-MS spectrum of BTC980.

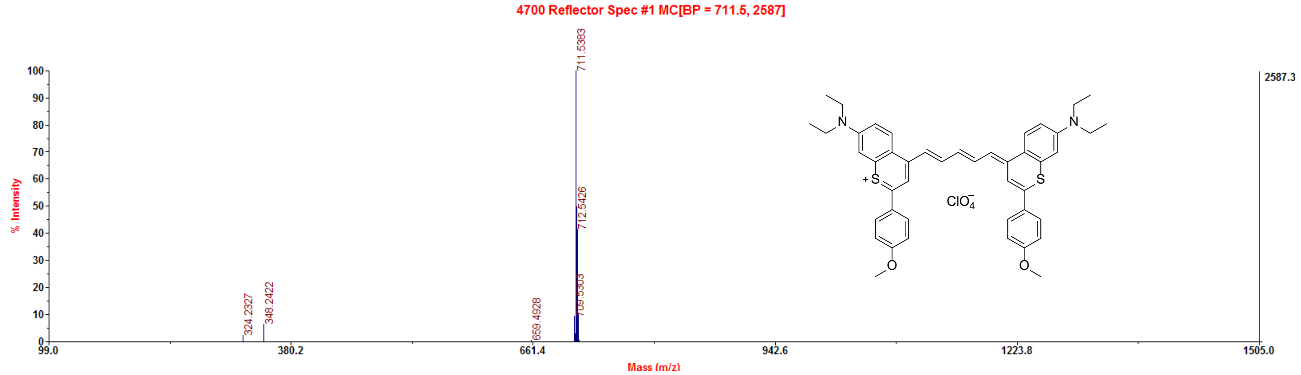

Supplementary Figure 51. Maldi-Tof/Tof-MS spectrum of BTC982.

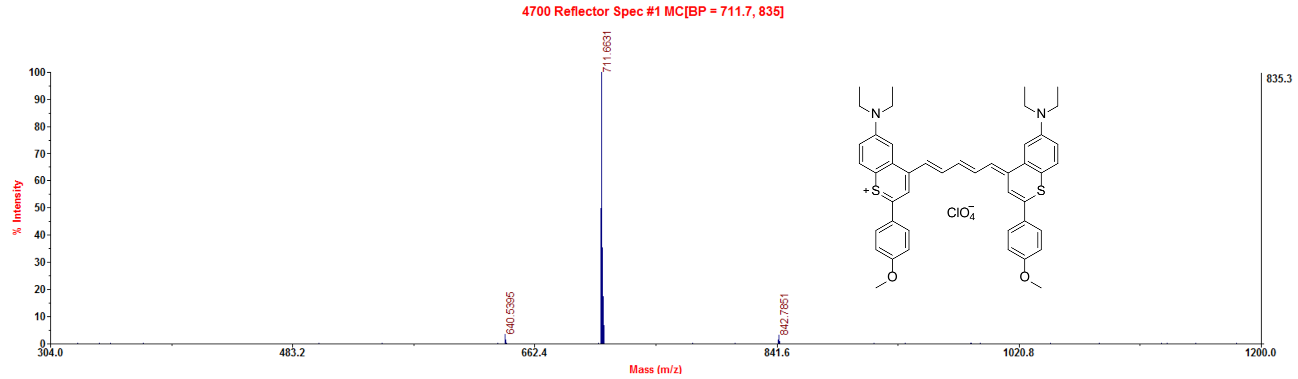

Supplementary Figure 52. Maldi-Tof/Tof-MS spectrum of BTC1070.

Supplementary Tables

Supplementary Table 1. Details of imaging parameters used in each fluorescent image of this study.

Figures	Dyes	Concentration/Dose	Laser (nm/mWcm^{-2})	Filter sets	Exposure time (ms)
**Fig. 1k** (inset)	BTCs, IR26	10 µM	915/~100	850LP, 1000LP	20
**Fig. 1k** (inset)	ICG	10 µM	808/~100	850LP, 1000LP	20
**Fig. 3b**	BTC1070, IR26	200 µM	1064/~100	1100LP, 1200LP	**Supplementary Table 2**
**Fig. 3b**	ICG (NIR-I)	100 µM	808/~100	850LP, 950SP	
**Fig. 3b**	ICG (NIR-II)	100 µM	808/~100	850LP, 1000LP	
**Fig. 4c**	IR26	10 nmol	1064/~60	1100LP, 1200LP	200
**Fig. 4d**, **4i**	ICG	5 nmol	808/~10	850LP, 950SP	50
**Fig. 4e**	ICG	5 nmol	808/~10	850LP, 1000LP	50
**Fig. 4f**, **4g**, **4k**, **Supplementary Fig. 17**	BTC1070	10 nmol	1064/~60	1100LP, 1200LP	200
**Fig. 6a** (red channel)	BTC1070	200 µM	808/~250	900LP	**Supplementary Table 2**
**Fig. 6a** (cyan channel)	BTC1070	200 µM	808/~250	900LP, 1000LP	
**Fig. 7a-c**, **Supplementary Fig. 25-27** (red channel)	BTC1070	10 nmol	808/~200	900LP	50
**Fig. 7a-c**, **Supplementary Fig. 25-27** (cyan channel)	BTC1070	10 nmol	808/~200	900LP, 1000LP	50
**Supplementary Fig. 10** (1100LP)	BTC1070	200 µM	1064/~100	1100LP	**Supplementary Table 2**
**Supplementary Fig. 10** (1200LP)	BTC1070	200 µM	1064/~100	1100LP, 1200LP	
**Supplementary Fig. 11** (1100LP)	BTC1070	200 µM	915/~100	1000LP, 1100LP	
**Supplementary Fig. 11** (1200LP)	BTC1070	200 µM	915/~100	1000LP, 1100LP, 1200LP	
**Supplementary Fig. 15**	BTC982	1.25 nmol	915/~4	850LP, 1000LP	5, 25, 50, 100
**Supplementary Fig. 16**	BTC982	1.25 nmol	915/~4	850LP, 1000LP	50

Supplementary Table 2. Details of exposure times (ms) used for each fluorescent image of tissue phantom imaging.

Depth (mm)	BTC1070						IR26	ICG	
	915 nm Ex		1064 nm Ex		808 nm Ex		1064 nm Ex	808 nm Ex	
	1100LP	1200LP	1100LP	1200LP	900LP	1000LP	1200LP	NIR-I[a]	NIR-II[a]
0	20	40	10	20	10	10	20	40	20
1	40	80	20	40	10	10	40	80	40
2	80	160	40	80	20	20	80	160	80
3	160	320	80	160	40	40	160	320	160
4	320	640	160	320	80	80	320	640	320
5	640	1280	320	640	N/A	N/A	N/A	N/A	N/A
6	1280	2560	640	1280	N/A	N/A	N/A	N/A	N/A
7	2560	5120	640	2560	N/A	N/A	N/A	N/A	N/A
8	N/A	N/A	640	5120	N/A	N/A	N/A	N/A	N/A

Exposure times were extending for each image acquired at deeper penetration depth to achieve sufficient signal and best signal-to-noise ratio. [a]NIR-I and NIR-II of ICG denote 850-950 nm and 1000-1700 nm region, respectively.

Supplementary Table 3. Photophysical Properties of IR26, BTC980, BTC982 and BTC1070 in different solvents.

Dye	Solvent[a]	λ_{abs} (nm)	ε (M^{-1} cm^{-1})	λ_{fl} (nm)	Stokes Shift (nm)	Φ_{fl}[b] (%)	$\varepsilon\Phi_{fl}$ (M^{-1} cm^{-1})
IR26	DCE	1084	130000	1144	60	0.05	65
	DCM	1073	150000	1138	65	0.03	46
	DMSO	1077	30000	1124	47	0.06	18
	MeOH	441	N/A	N/A	N/A	N/A	N/A
	PBS	1080	3750	1095	15	0.01	0.375
BTC980	DCM	932	184000	980	48	0.57	1049
	$CHCl_3$	939	186000	982	43	0.76	1414
	DMSO	924	106000	972	48	1.4	1484
	MeOH	908	80000	950	42	0.3	240
	PBS	920	79600	950	30	0.22	175
BTC982	DCM	944	260000	982	38	0.68	1768
	$CHCl_3$	944	297000	984	40	0.81	2406
	DMSO	948	200000	986	38	1.26	2520
	MeOH	927	241000	962	35	0.41	988
	PBS	950	137500	988	38	0.3	412
BTC1070	DCM	1014	115000	1070	56	0.09	104
	$CHCl_3$	1005	133000	1054	49	0.16	213
	DMSO	1012	79000	1066	54	0.04	32
	MeOH	989	97000	1042	53	0.02	19
	PBS	1015	45000	1065	50	0.016	7.2

[a]Samples in water were all based on the phospholipid micelle formulations with a dye loading capacity of ~1wt%. [b]For determination of the fluorescence quantum efficiency (Φ_{fl}), IR-26 in dichloroethane (Φ_{fl} = 0.05%) was used as a fluorescence standard.[4] DCM: dichloromethane, DCE: dichloroethane, $CHCl_3$: chloroform, DMSO: dimethyl sulfoxide, MeOH: methanol, PBS: phosphate buffer.

Supplementary Table 4. Comparison of attenuation coefficients (τ) of capillary signals under 915 and 1064 nm excitation.

Filter	τ	
	915 nm Ex	1064 nm Ex
1100LP	0.7920	0.6944
1200LP	0.7946	0.6662

Data were derived from **Supplementary Figure 10** and **11**. 1100LP: 1100-1700 nm, 1200LP: 1200-1700 nm

Supplementary Table 5. SBR and FWHM of capillary images of BTC1070 under 915 and 1064 nm excitation.

Depth (mm)	Filter	SBR		FWHM	
		915 nm Ex	1064 nm Ex	915 nm Ex	1064 nm Ex
1	1100LP	40.95 ± 7.42	41.55 ± 6.51	0.53 ± 0.01	0.49 ± 0.01
	1200LP	41.75 ± 4.84	71.83 ± 11.67	0.51 ± 0.01	0.55 ± 0.01
2	1100LP	10.58 ± 0.98	11.13 ± 2.81	0.72 ± 0.17	0.65 ± 0.03
	1200LP	16.43 ± 2.60	20.41 ± 4.25	0.56 ± 0.03	0.54 ± 0.06
3	1100LP	5.51 ± 0.63	6.23 ± 1.46	1.90 ± 0.11	1.66 ± 0.17
	1200LP	8.96 ± 0.87	11.00 ± 1.40	1.04 ± 0.05	0.72 ± 0.09
4	1100LP	3.76 ± 0.20	3.69 ± 0.21	2.98 ± 0.17	2.71 ± 0.17
	1200LP	5.03 ± 1.04	5.74 ± 0.51	1.28 ± 0.15	0.93 ± 0.17
5	1100LP	2.60 ± 0.14	2.80 ± 0.20	4.06 ± 0.17	3.70 ± 0.11
	1200LP	3.18 ± 0.39	4.40 ± 0.55	2.58 ± 0.15	2.24 ± 0.30

Data were derived from **Supplementary Figure 10** and **11**. 1100LP: 1100-1700 nm, 1200LP: 1200-1700 nm

Through longitudinal comparison, the SBRs and FWHMs presented in **Supplementary Table 5** show emission wavelength dependency at various depths, which can be attributed to the reduced photo scattering at longer wavelength. Through crosswise comparison, the SBRs and FWHMs presented in **Supplementary Table 5** show excitation wavelength dependency, which can be attributed to the lower signal attenuation coefficient of 1064 nm excitation.

Supplementary Table 6. The experimental and calculated ratios and the corresponding coefficient of variation (CV) for each pH group.

Depth (mm)	Experimental ratios				Calculated ratios[a]			
	pH = 1.15	pH = 2.17	pH = 3.22	pH = 4.35	pH = 1.15	pH = 2.17	pH = 3.22	pH = 4.35
0	0.5444	0.5957	0.7593	0.8654	0.5444	0.5957	0.7593	0.8654
1	0.5570	0.6188	0.8002	0.8608	0.5322	0.5909	0.7498	0.8807
2	0.5632	0.6394	0.7737	0.8727	0.5202	0.5862	0.7403	0.8963
3	0.5547	0.5988	0.7538	0.8825	0.5084	0.5814	0.7310	0.9121
4	0.5367	0.5735	0.7330	0.8579	0.4970	0.5768	0.7217	0.9282
CV (%)	1.91	4.12	3.27	1.14	3.60	1.28	2.01	2.77

[a] Based on **Supplementary Equation 13** (**Supplementary Note 2**), where r_0 are the ratio values at 0 mm depth for each group and $\Delta\tau$ are derived from the single-exponential fitting curves in Figure 6c. $|\Delta\tau| \geq 0.0317$ will lead to CVs higher than 5%.

Supplementary Table 7. pH values resolved from ratiometric images and measured by pH meter.

Analysis mode	pH Values[a]		
	Low pH group	High pH group	Normal pH group
noninvasive	1.92 ± 0.41	2.86 ± 0.25	3.70 ± 0.10
invasive	2.00 ± 0.08	2.87 ± 0.22	3.68 ± 0.20
gastric fluid	2.16 ± 0.07	3.10 ± 0.16	3.80 ± 0.12
pH meter	2.10 ± 0.11	3.08 ± 0.27	3.95 ± 0.18[b]

[a]Ratios derived from the ratio images (**Figure 7d and Supplementary Figure 25-27**) were converted to pH values by means of **Supplementary Equation 14-18** (**Supplementary Note 3**). [b]The pH values were measured from the mice (n = 3) after gavage of fluorescent probes, which are independent to the normal pHs measured (n = 6) in the method section.

Supplementary Methods

Synthesis of compound 1d. Compound **1b** was synthesized according to the literature by a microwave-assisted three-component coupling-addition-S_NAr method.[1] ^{1}H NMR (400 MHz, $CDCl_3$) δ 8.55 (d, *J* = 8.0 Hz, 1H), 7.66 (m, 8.3 Hz, 4H), 7.57 (t, *J* = 7.4 Hz, 1H), 7.34 (s, 1H), 7.02 (d, *J* = 8.7 Hz, 2H), 3.88 (s, 3H).

To a solution of compound **1b** (536 mg, 2 mmol) in anhydrous THF (10 mL) was added dropwise 1.0 M CH_3MgBr (6 mL). After stirring at room temperature for 2 h under nitrogen, the solution was poured into 10% aqueous $HClO_4$ (20 mL) and allowed to stir for 5 min before the solid was isolated by filtration. The resulting solid was dried to give **1d** (yield: 700 mg, 95%) as an orange red solid. ^{1}H NMR (400 MHz, CD_3CN) δ 8.79 – 8.63 (m, 2H), 8.48 (d, *J* = 8.0 Hz, 1H), 8.25 – 8.06 (m, 4H), 7.24 (d, *J* = 8.7 Hz, 2H), 3.96 (s, 3H), 3.18 (s, 3H); ^{13}C NMR (101 MHz, CD_3CN) δ 175.5, 167.4, 166.4, 141.6, 135.4, 133.6, 132.4, 131.8, 131.0, 130.1, 129.4, 127.5, 117.3, 57.1, 24.4; Maldi-Tof/Tof-MS: calcd for $C_{17}H_{15}OS^+$ $[M]^+$, 267.0844; Found, 267.0746. $[M]^+$.

Synthesis of BTC980. To a suspension of N-[3-(phenylamino)allylidene]aniline monohydrochloride (64.7 mg, 0.25 mmol) in MeCN (2 mL) was added NaOAc (41 mg, 0.5 mmol) and Ac_2O (1 mL). The mixture was stirred at ambient temperature for 30 min. Compound **1d** (183.4 mg, 0.5 mmol) were added to the resulting clear solution and subsequently heated at 100 ºC under nitrogen for 3 h. After cooling to room temperature, the mixture was evaporated and purified by a flash column chromatography [silica gel, MeOH/DCM = 1/100, v/v] to give **BTC980** (yield: 100 mg, 60%). ^{1}H NMR (400 MHz, DMSO-TFA) δ 8.45 (d, *J* = 7.1 Hz, 4H), 8.24 (s, 2H), 7.86 (d, *J* = 8.1 Hz, 6H), 7.70 – 7.51 (m, 6H), 7.16 (m, 1H), 7.09 (d, *J* = 8.8 Hz, 4H), 3.85 (s, 6H); Maldi-Tof/Tof-MS: calcd for $C_{37}H_{29}O_2S_2^+$ $[M]^+$, 569.1609; Found, 569.0539. $[M]^+$.

Synthesis of compound 2d. A mixture of polyphosphoric acid (PPA, 22 g), 3-bromothiophenol (2 g, 10.6 mmol) and ethyl 3-(4-methoxyphenyl)-3-oxopropanoate (2.6 g, 11.66 mmol) was stirred at 95 ºC for 2 h. After cooling to room temperature, ice water was added to quench the reaction and extracted with DCM (100 mL × 3). The combined organic extracts were dried with Na_2SO_4, filtered, and evaporated. The crude product was further purified by a flash column chromatography [silica gel, PE/DCM = 1/1 to 0/1, v/v] to give **2b** (yield: 2.6 g , 70%). ^{1}H NMR (400 MHz, $CDCl_3$) δ 8.37 (d, *J* = 8.6 Hz, 1H), 7.80 (s, 1H), 7.64 (d, *J* = 8.5 Hz, 2H), 7.26 (s, 1H), 7.20 (s, 1H), 7.01 (d, *J* = 8.7 Hz, 2H), 3.88 (s, 3H); ^{13}C NMR (100 MHz, $CDCl_3$) δ 180.3, 162.2, 152.9, 139.3, 135.4, 131.3, 130.3, 129.6, 128.8, 128.5, 126.8, 122.2, 114.9, 55.7; Maldi-Tof/Tof-MS: calcd for $C_{16}H_{12}BrO_2SH^+$ $[M+H]^+$, 346.9741; Found, 347.0120. $[M+H]^+$.

A tube was charged with compound **2b** (347 mg, 1 mmol), $Pd_2(dba)_3$ (22.3 mg, 0.025 mmol), DavePhos (9.8 mg, 0.025 mmol), and Cs_2CO_3 (815 mg, 2.5 mmol, 2.5 eq). The tube was sealed and evacuated/backfilled with nitrogen (3×). Anhydrous dioxane (5 mL) was added, following the addition of diethylamine (520 µL, 5 mmol, 5 eq). The reaction was stirred at 100 °C for 18 h. After cooling to room temperature, the mixture was filtered, and the filtrate was evaporated. The crude product was further purified by a flash column chromatography [silica gel, PE/DCM = 1/1 to 0/1, v/v, then EA/DCM = 1/10, v/v] to give **2c** (yield: 288 mg, 85%). ^{1}H NMR (400 MHz, $CDCl_3$) δ 8.33 (d, *J* = 9.2 Hz, 1H), 7.63 (dd, *J* = 6.9, 1.9 Hz, 2H), 7.05 (s, 1H), 7.01 – 6.97 (m, 2H), 6.84 (dd, *J* = 9.2, 2.4 Hz, 1H), 6.66 (d, *J* = 2.4 Hz, 1H), 3.87 (s, 3H), 3.45 (q, *J* = 7.1 Hz, 4H), 1.23 (t, *J* = 7.1 Hz, 6H); ^{13}C NMR (100 MHz, $CDCl_3$) δ 180.4, 161.6, 150.6, 149.7, 140.5, 130.1, 129.5, 128.3, 122.2, 119.8, 114.6, 112.9, 104.8, 55.5, 44.7, 12.7; Maldi-Tof/Tof-MS: calcd for $C_{20}H_{22}NO_2SH^+$ $[M+H]^+$, 340.1371; Found, 340.1707. $[M+H]^+$.

Compound **2c** (200 mg, 0.59 mmol) was dissolved in anhydrous THF (5 mL) in a flame dried flask under nitrogen. 1.0 M CH_3MgBr (1.8 mL) was added dropwise to this solution and allowed to stir at room temperature for 2 h. The solution was poured into 10% aqueous $HClO_4$ (10 mL) and extracted with DCM (25 mL × 3). The combined organic extracts

were dried with Na_2SO_4, filtered, and evaporated to give **2d** (yield: 250 mg, 97%) without further purification. ^{1}H NMR (400 MHz, Acetone-D6) δ 8.54 (d, *J* = 9.6 Hz, 1H), 8.27 (s, 1H), 8.06 (d, *J* = 8.3 Hz, 2H), 7.70 (d, *J* = 17.0 Hz, 2H), 7.24 (d, *J* = 8.2 Hz, 2H), 3.96 (s, 3H), 3.83 (d, *J* = 7.1 Hz, 4H), 3.06 (s, 3H), 1.35 (t, *J* = 6.8 Hz, 6H); ^{13}C NMR (100 MHz, Acetone-D6) δ 165.1, 163.1, 161.7, 153.0, 147.0, 133.4, 130.5, 127.9, 125.8, 122.4, 120.6, 116.5, 106.4, 56.4, 46.4, 23.0, 12.8; Maldi-Tof/Tof-MS: calcd for $C_{21}H_{24}NOS^+$ $[M]^+$, 338.1579; Found, 338.2363. $[M]^+$.

Synthesis of compound 3d. Compound **3b** was synthesized by the similar procedure as compound 2b (yield: 1.5 g, 41%). ^{1}H NMR (400 MHz, $CDCl_3$) δ 8.63 (s, 1H), 7.68 (d, *J* = 8.2 Hz, 1H), 7.61 (d, *J* = 8.4 Hz, 2H), 7.49 (d, *J* = 8.5 Hz, 1H), 7.18 (s, 1H), 6.99 (d, *J* = 8.4 Hz, 2H), 3.86 (s, 3H); ^{13}C NMR (100 MHz, $CDCl_3$) δ 179.7, 162.2, 153.2, 136.5, 134.8, 132.3, 131.4, 128.6, 128.5, 128.2, 122.2, 122.1, 114.9, 55.8; Maldi-Tof/Tof-MS: calcd for $C_{16}H_{12}BrO_2SH^+$ $[M+H]^+$, 346.9741; Found, 346.9849. $[M+H]^+$.

Compound **3c** was synthesized by the similar procedure as compound 2c (yield: 280 mg, 83%). ^{1}H NMR (400 MHz, $CDCl_3$) δ 7.72 (d, *J* = 2.7 Hz, 1H), 7.66 – 7.62 (m, 2H), 7.47 (d, *J* = 8.9 Hz, 1H), 7.16 (s, 1H), 7.04 (d, *J* = 7.5 Hz, 1H), 7.00 – 6.95 (m, 2H), 3.86 (s, 3H), 3.46 (q, *J* = 7.1 Hz, 4H), 1.21 (t, *J* = 7.1 Hz, 6H); ^{13}C NMR (100 MHz, $CDCl_3$) δ 181.2, 161.7, 152.4, 147.7, 132.2, 129.6, 128.4, 127.7, 123.7, 121.2, 117.4, 114.7, 108.2, 55.7, 44.7, 12.7; Maldi-Tof/Tof-MS: calcd for $C_{20}H_{22}NO_2SH^+$ $[M+H]^+$, 340.1371; Found, 340.1465. $[M+H]^+$.

Compound **3d** was synthesized by the similar procedure as compound 2d (yield: 250 mg, 97%). ^{1}H NMR (400 MHz, Acetone-D6) δ 8.77 (s, 1H), 8.43 (d, *J* = 9.2 Hz, 1H), 8.18 (d, *J* = 8.0 Hz, 2H), 7.83 (d, *J* = 9.0 Hz, 1H), 7.44 (s, 1H), 7.25 (d, *J* = 8.1 Hz, 2H), 3.98 (s, 3H), 3.74 (d, *J* = 6.6 Hz, 4H), 3.13 (s, 2H), 1.32 (t, *J* = 6.8 Hz, 6H); ^{13}C NMR (100 MHz, Acetone-D6) δ 166.0, 165.7, 160.7, 151.4, 134.1, 130.9, 130.5, 130.4, 129.4, 127.7, 122.8, 116.8, 105.4, 56.5, 45.6, 24.1, 12.2; Maldi-Tof/Tof-MS: calcd for C21H24NOS$^+$ $[M]^+$, 338.1579; Found, 338.2362. $[M]^+$.

Synthesis of BTC982. To a suspension of N-[3-(phenylamino)allylidene]aniline monohydrochloride (64.7 mg, 0.25 mmol) in MeCN (2 mL) was added NaOAc (41 mg, 0.5 mmol) and Ac_2O (1 mL). The mixture was stirred at ambient temperature for 30 min. Compound 2d (219 mg, 0.5 mmol) were added to the resulting clear solution and subsequently heated at 100 °C under nitrogen for 3 h. After cooling to room temperature, the mixture was evaporated and purified by a flash column chromatography [silica gel, MeOH/DCM = 0/100 to 1/100, v/v] to give **BTC982** (yield: 96 mg, 47%). ^{1}H NMR (600 MHz, CD_3CN) δ 7.48 (t, *J* = 11.8 Hz, 4H), 7.27 (d, *J* = 8.4 Hz, 4H), 7.23 (s, 2H), 6.78 (d, *J* = 8.4 Hz, 4H), 6.61 (d, *J* = 13.2 Hz, 2H), 6.47 (t, *J* = 12.2 Hz, 1H), 6.42 (dd, *J* = 9.1, 2.3 Hz, 2H), 6.33 (d, *J* = 2.4 Hz, 2H), 3.78 (s, 6H), 3.27 (q, *J* = 7.1 Hz, 8H), 1.09 (t, *J* = 7.3 Hz, 12H); ^{13}C NMR (100 MHz, CD_3CN) δ 162.4, 149.6, 147.3, 146.2, 145.3, 139.8, 132.3, 129.1, 128.8, 128.5, 121.2, 117.7, 116.6, 115.2, 106.4, 56.1, 45.3, 13.1; Maldi-Tof/Tof-MS: calcd for $C_{45}H_{47}N_2O_2S_2^+$ $[M]^+$, 711.3079; Found, 711.5383. $[M]^+$.

Synthesis of BTC1070. BTC1070 was synthesized by the similar procedure as BTC982 (yield: 55 mg, 27%). ^{1}H NMR (500 MHz, DMSO-TFA) δ 8.37 (t, *J* = 12.7 Hz, 2H), 8.21 (s, 2H), 7.90 (d, *J* = 8.5 Hz, 4H), 7.83 (d, *J* = 7.9 Hz, 2H), 7.24-7.56 (m, 7H), 7.17 (d, *J* = 8.8 Hz, 4H), 3.88 (s, 6H), 3.56 (q, *J* = 7.0 Hz, 8H), 1.18 (t, *J* = 7.0 Hz, 12H); Maldi-Tof/Tof-MS: calcd for $C_{45}H_{47}N_2O_2S_2^+$ $[M]^+$, 711.3079; Found, 711.6631. $[M]^+$. Note that a little D-TFA in DMSO could improve the splitting of ^{1}H NMR signals on aromatic protons without protonation.

Preparation of BTCs-loaded phospholipid nanomicelles. The nanomicelles were prepared using modified film hydration technique. In a typical procedure, BTCs were firstly dissolved in $CHCl_3$ to obtain stock solution with concentration of 5 mM. 100 μL (0.5 μmol) stock solution of BTCs was mixed with 1.6 mL DOPE-mPEG2000 (25 mg/mL in $CHCl_3$) at a mass ratio of ~1:100. The solvent was removed by vacuum rotary evaporation to form a dry dye-containing lipid film. The dried film was hydrated with 10 mL deionized water at 60 °C and sonication for 30 seconds to make the clear

nanomicelles solution with dye concentration of 50 μM. The nanomicelle solution was further concentrated if necessary by using a 30 K Amicon Ultra filter (Millipore Corporation) under centrifugation at 2,000 g for 5 min.

Determination of fluorescence quantum yields. Quantum yields (Φ_{fl}) were determined in various solvents relative to IR26 (Φ_{fl} = 0.05% in DCE), from plots of integrated fluorescence intensity vs. absorbance, according to the following relationship:

$$\Phi_{\mathrm{fl,s}} = \Phi_{\mathrm{fl,r}} \times \frac{n_{\mathrm{s}}^2}{n_{\mathrm{r}}^2}\left(\frac{K_{\mathrm{s}}}{K_{\mathrm{r}}}\right) \quad (1)$$

where subscripts r and s denote standard and test sample, respectively, Φ_{fl} is the fluorescence quantum yield, K is the slop of the integrated fluorescence intensity vs. absorbance plot, and n is the refractive index of the solvent. Measurements were performed with the absorbance at 808 nm of all dye solutions ≤ 0.08 in order to maximize illumination homogeneity and optical transparency. The 808 nm laser was used as the excitation source and the emission spectrum in the 900-1500 nm region was acquired in fluorescence spectrometer. For determination the quantum yields of BTCs-loaded nanomicelles in aqueous solution, excitation values and emission collection were as follows: BTC980 vs. IR26 (860 nm Ex, collection 900-1500 nm), BTC982 vs. IR26 (915 nm Ex, collection 930-1500 nm), BTC1070 vs. IR26 (940 nm Ex, collection 1000-1500 nm).

Quantum Calculations. All the quantum chemical calculations were done with the Gaussian 09 suite.[2] The geometry optimizations of the fluorophores were performed using density functional theory (DFT) with Becke's three-parameter hybrid exchange function with Lee−Yang−Parr gradient-corrected correlation functional (B3-LYP functional) and 6-31G(d) basis set. No constraints to bonds/angles/dihedral angles were applied in the calculations, and all atoms were free to optimize. The electronic transition energies and corresponding oscillator strengths were calculated with time-dependent density functional theory (TDDFT) at the B3LYP/6-311G(d,p) level.

Brightness comparison of BTCs, IR26 and ICG. Recent reports have demonstrated NIR-I indocyanine green (ICG) fluorophores have bright NIR-II tail emission.[3] For example, the brightness of ICG in NIR-II region is roughly 9 times brighter than IR-E1050 (a commercial donor-acceptor-donor-type NIR-II fluorophore) in water. Therefore, BTCs and IR26 were benchmarked against the emission brightness of ICG in PBS (pH 7.4) at equimolar concentration of 10 μM. The solutions were filled in capillaries (ϕ = 1 mm) and imaged individually in the above-described setup with 808-nm (for ICG)/915-nm (for BTCs and IR26) excitation at same fluence rate of ~100 mW cm^{-2}. The emissions were all filtrated through two filters of 850 nm and 1000 nm longpass and then collected at exposure time of 20 ms. In the resulting background-corrected images, the average emission intensity was calculated from a region of interest within the capillaries.

Determination of p*K*a values. The calculation of p*K*a values was performed with Origin 2017 software (OriginLab, Northhampton, MA), using the Bolzmman fitting function (**Supplementary Equation 2**):

$$y = \frac{A_1 - A_2}{1+\mathrm{e}^{(x-x_0)/dx}} + A_2 \quad (2)$$

On the basis of the calculated equation of p*K*a, x_0 corresponds to the p*K*a value.

Cell viability. CaOV3 cell line was provided by American Type Culture Collection (ATCC, Manassas, VA, USA). The cytotoxicity was measured by using Cell Counting Kit-8 (CCK-8) assay. The cells (1×10^4) were incubated in each well of a 96-well plate for 24 h, then incubated with BTC982 and BTC1070 micelles (dye concentration: 0, 5, 10, 15, 20, 25,

30, 40, 50 μM) with different concentrations for 24 h, respectively. Enzyme dehydrogenase in living cells was oxidized by this kit to orange carapace. The quality was assessed calorimetrically by using a multi-reader (TECAN, Infinite M200, Germany). The measurements were based on the absorbance values at 450 nm. Following formula was used to calculate the viability of cell growth: Viability (%) = (mean absorbance value of treatment group/mean absorbance value of control group) × 100.

Supplementary Notes

Supplementary Note 1. Mathematical description for the light/Intralipid interaction in tissue phantom imaging.

To accurately describe and calculate the fluorescence signal changes in tissue is difficult, because of the complicated and wavelength-dependent light propagation in inhomogenous tissue. Here, we assumed that only scattering and absorption contribute to the loss of excitation and emission light energy, resulting in fluorescence signal attenuation in tissue. According to a proposed mathematical equation[5], the optical irradiance (E_d, mW cm^{-2}) propagating through 1% Intralipid is given by

$$E_d = E_0 \mathrm{e}^{[-(\mu_\mathrm{a}+\mu_\mathrm{s})d]} \tag{3}$$

where E_0 is the incident irradiance, μ_a and μ_s are, respectively, the absorption and scattering coefficients of Intralipid to the incident irradiance, d is the geometrical path length (or penetration depth) in Intralipid. The above **Supplementary Equation 3** could be utilized to describe the interaction of excitation laser with Intralipid. Likewise, fluorescence emission after propagating through 1% Intralipid (F_d) is given by

$$F_d = F_0' \mathrm{e}^{[-(\mu_\mathrm{a}'+\mu_\mathrm{s}')d]} \tag{4}$$

where F_0' is the fluorescence emission before propagating through 1% Intralipid, μ_a' and μ_s' are, respectively, the absorption and scattering coefficients of Intralipid to the fluorescence emission. The excitation and emission irradiance have the following relationships

$$F_0 = E_0 \Phi_\mathrm{fl} \tag{5}$$

$$F_0' = E_d \Phi_\mathrm{fl} \tag{6}$$

Where F_0 is the fluorescence emission at 0 mm depth, Φ_fl is the fluorescence quantum yield. By combining **Supplementary Equation 3-6**, the relationship between fluorescence intensity and penetration depth can be given by

$$F_d = F_0 \mathrm{e}^{[-(\mu_\mathrm{a}+\mu_\mathrm{s}+\mu_\mathrm{a}'+\mu_\mathrm{s}')d]}$$

$$F_d = F_0 \mathrm{e}^{[-(\mu_\mathrm{t}+\mu_\mathrm{t}')d]} \tag{7}$$

where $\mu_\mathrm{t} = \mu_\mathrm{a} + \mu_\mathrm{s}$, $\mu_\mathrm{t}' = \mu_\mathrm{a}' + \mu_\mathrm{s}'$ denote the total attenuation coefficients of excitation and emission light, respectively. The result is in accordance with the Lambert-Beer law, where signal attenuation depends exponentially on the depth (*d*) of an emitting structure and the attenuation coefficient of the medium to excitation (μ_t) and emission (μ_t') photons. Therefore, signal attenuation coefficients derived from the fitting curves were contributed equally by the attenuation of excitation and emission light, $\tau = \mu_\mathrm{t} + \mu_\mathrm{t}'$, so that

$$F_d = F_0 \mathrm{e}^{-\tau d} \tag{8}$$

The result reveals the excitation and emission wavelength-dependent signal attenuation with respect to penetration depth.

Supplementary Note 2. Mathematical description for the ratio changes in tissue phantom imaging.

According to **Supplementary Equation 8**, fluorescence intensity at 900-1700 nm is given by

$$F_{1(d)} = F_{1(0)}\mathrm{e}^{-\tau_1 d} \tag{9}$$

where $F_{1(d)}$, $F_{1(0)}$ are fluorescence intensities of capillary at 0 and d mm depth, respectively. Likewise, for fluorescence intensity at 1000-1700 nm, there is

$$F_{2(d)} = F_{2(0)}\mathrm{e}^{-\tau_2 d} \tag{10}$$

where $F_{2(d)}$, $F_{2(0)}$ are fluorescence intensities of capillary at 0 and d mm depth, respectively. Ratiometric fluorescence signals at 0 mm (r_0) and d mm (r_d) depth are, respectively, given by

$$r_0 = \frac{F_{2(0)}}{F_{1(0)}} \tag{11}$$

$$r_d = \frac{F_{2(d)}}{F_{1(d)}} \tag{12}$$

Substituting **Supplementary Equation 9**, **10** and **11** into **Supplementary Equation 12**, we obtained the following equation

$$r_d = r_0\mathrm{e}^{(\tau_1-\tau_2)d} \tag{13}$$

Therefore, it can be concluded that the difference of attenuation coefficient between 900-1700 nm and 1000-1700 nm, $\Delta\tau = \tau_1 - \tau_2$, determines the ratiometric fluorescence signal changes with increased depth.

Supplementary Note 3. Resolving pH from in vivo ratiometric fluorescence imaging.

Five calibration functions for different depth (0, 1, 2, 3, 4 mm) derived from the curve fitting in **Figure 6d** were, respectively, given by

0 mm depth: $$r_0 = \frac{-0.3197}{1+\mathrm{e}^{(\mathrm{pH}-2.9929)/0.4043}} + 0.876 \quad r^2 = 0.980 \tag{14}$$

1 mm depth: $$r_1 = \frac{-0.3088}{1+\mathrm{e}^{(\mathrm{pH}-2.7039)/0.3861}} + 0.8648 \quad r^2 = 0.998 \tag{15}$$

2 mm depth: $$r_2 = \frac{-0.3662}{1+\mathrm{e}^{(\mathrm{pH}-2.8397)/0.6616}} + 0.9063 \quad r^2 = 0.999 \tag{16}$$

3 mm depth: $$r_3 = \frac{-0.3554}{1+\mathrm{e}^{(\mathrm{pH}-3.0828)/0.484}} + 0.9066 \quad r^2 = 0.999 \tag{17}$$

4 mm depth: $$r_4 = \frac{-0.3453}{1+\mathrm{e}^{(\mathrm{pH}-3.0692)/0.4526}} + 0.8771 \quad r^2 = 0.999 \tag{18}$$

where r_0, r_1, r_2, r_3 and r_4 denote ratios at varying depth of 0, 1, 2, 3 and 4 mm, respectively. The above calibration functions were used to convert the ratios to pHs according to the measured tissue thickness in **Supplementary Figure 24**. Specifically, **Supplementary Equation 14** was used for pH calibration in the ratiometric imaging of exposed gastric fluid. **Supplementary Equation 15** was used for pH calibration in invasive ratiometric imaging of gastric fluid wrapped in a thin gastric wall (1.04 ± 0.25 mm). **Supplementary Equation 16-18** were used for pH calibration in non-invasive ratiometric imaging of gastric fluid at a tissue depth of ~2-4 mm (composed of gastric wall, muscle, fat and skin). pHs measured by ratiometric imaging and pH meter were summarized in the **Supplementary Table 7**.

Supplementary References

(1) Willy B, Frank W, Muller TJJ. Microwave-assisted three-component coupling-addition-SNAr (CASNAR) sequences to annelated 4H-thiopyran-4-ones. *Organic & Biomolecular Chemistry* **8**, 90-95 (2010).

(2) Frisch, M, *et al*. Gaussian 09, revision D.01; Gaussian, Inc.: Wallingford, CT (2009).

(3) Carr JA*, et al.* Shortwave infrared fluorescence imaging with the clinically approved near-infrared dye indocyanine green. *Proc. Natl. Acad. Sci. USA* **115**, 4465-4470 (2018).

(4) Semonin OE, Johnson JC, Luther JM, Midgett AG, Nozik AJ, Beard MC. Absolute Photoluminescence Quantum Yields of IR-26 Dye, PbS, and PbSe Quantum Dots. *J. Phys. Chem. Lett.* **1**, 2445-2450 (2010).

(5) van Staveren HJ, Moes CJM, van Marie J, Prahl SA, van Gemert MJC. Light scattering in lntralipid-10% in the wavelength range of 400–1100 nm. *Appl. Opt.* **30**, 4507-4514 (1991).
